# Pin1 targets phosphorylated NCOA4 for K29/K48-linked ubiquitination to suppress ferritinophagy and ferroptosis in anaplastic thyroid cancer

**DOI:** 10.1038/s41419-026-09058-5

**Published:** 2026-07-01

**Authors:** Zicheng Sun, Fan Bai, Mingwei Liang, Hannah Xiaoyan Hui, Huazhen Xu, Zhaoxi Zhang, Xin Liu, Peng Hu, Weiming Lv, Rui Ge, Jie Li

**Affiliations:** 1 Department of Thyroid Surgery, The First Affiliated Hospital, Sun Yat-sen University, Guangzhou, China; 2 Department of Head and Neck Surgery, Guangdong Engineering Research Center of AI-Powered Precision Cancer Diagnostics and Therapeutics (Proposed)/ Guangdong Engineering Technology Research Center of AI-Powered Precision Cancer Diagnostics and Therapeutics, Cancer Hospital of Shantou University Medical College, Shantou, China; 3 Department of Oncology, The Sixth Affiliated Hospital, Sun Yat-sen University, Guangzhou, China; 4 School of Biomedical Sciences, The Chinese University of Hong Kong, Hong Kong SAR, China; 5 Scientific Research Center, The Seventh Affiliated Hospital, Sun Yat-sen University, Shenzhen, China; 6 Department of General Surgery, Huadong Hospital Affiliated to Fudan University, Shanghai, China

**Keywords:** Thyroid cancer, Cell death

## Abstract

Anaplastic thyroid cancer (ATC) is an aggressive endocrine malignancy characterized by rapid progression and limited therapeutic options. Ferritinophagy, a selective autophagic process mediated by NCOA4, regulates intracellular iron homeostasis. Here, we demonstrate that the prolyl isomerase Pin1 drives ATC progression by suppressing NCOA4-dependent ferritinophagy. Mechanistically, Pin1 recognizes phosphorylated NCOA4 through its substrate-binding WW domain. CDK2 phosphorylates NCOA4 at Ser572, creating a binding site for Pin1. This interaction promotes K29/K48-linked polyubiquitination and proteasomal degradation of NCOA4, thereby blocking ferritinophagy, lowering intracellular Fe²⁺ and lipid peroxidation, and ultimately suppressing ferroptosis. Pin1 knockdown or treatment with the pharmacological Pin1 inhibitor KPT-6566 stabilizes NCOA4, enhances ferritinophagy, and triggers ferroptosis, markedly restraining ATC growth both in vitro and in vivo. These findings identify Pin1 as a previously unrecognized suppressor of ferroptosis through NCOA4-dependent ferritinophagy and support Pin1 inhibition as a potential therapeutic strategy for ATC.

## Introduction

Thyroid cancer is one of the most common endocrine malignancies, and its incidence continues to rise worldwide. It encompasses four pathological types: papillary thyroid cancer (PTC), follicular thyroid cancer (FTC), anaplastic thyroid cancer (ATC), and medullary thyroid cancer (MTC) [1]. Among them, ATC is rare but extremely aggressive, with a median survival of less than six months [2–4]. Despite advances in understanding ATC biology, effective treatments remain lacking, underscoring the urgent need to uncover new molecular mechanisms and therapeutic targets. Recent evidence suggests that regulated cell death pathways may represent exploitable therapeutic vulnerabilities in aggressive and treatment-refractory cancers.

Ferroptosis is a regulated form of cell death driven by iron-dependent lipid peroxidation and excessive reactive oxygen species (ROS) accumulation [5–7]. This process is tightly regulated by cellular redox balance and iron metabolism [8]. Dysregulated ferroptosis contributes to tumor progression, as cancer cells develop multiple mechanisms to evade ferroptotic death, thereby sustaining growth and metastasis [9–12]. Ferritinophagy, mediated by the cargo receptor NCOA4, is a selective autophagic process that degrades ferritin to regulate intracellular iron availability [13]. This process releases ferrous iron from ferritin into the cytoplasm, fueling lipid peroxidation and triggering ferroptosis [14–16]. Accordingly, NCOA4-mediated ferritinophagy has been linked to multiple diseases, particularly cancer [17]. NCOA4 undergoes several post-translational modifications that critically modulate ferritinophagy [18–21]. E3 ubiquitin ligases such as TRIM7, HERC2, and DTX2 promote tumor ferroptosis resistance by mediating NCOA4 ubiquitination and degradation [18–20].

Pin1 is a key regulator of phosphorylation-dependent signaling that recognizes phosphorylated Ser/Thr-Pro motifs and modulates substrate conformation and function [22, 23]. Pin1 binds to phosphorylated substrates and promotes their ubiquitination and proteasomal degradation [24, 25]. Previous studies showed that Pin1 interacts with phosphorylated VHL and facilitates its ubiquitin-mediated degradation [25]. Therefore, Pin1 affects multiple oncogenic processes and is closely associated with tumor progression [24, 26–28]. However, whether Pin1 contributes to ferroptosis regulation through modulation of ferritinophagy remains unknown. Although Pin1 has been implicated in diverse phosphorylation-dependent signaling pathways, its role in ATC progression and iron-dependent cell death has not been fully elucidated.

In this study, we identify Pin1 as a critical suppressor of ferroptosis in ATC. Mechanistically, CDK2-mediated phosphorylation of NCOA4 at Ser572 creates a recognition motif for Pin1, promoting K29/K48-linked ubiquitination and proteasomal degradation of NCOA4. This process suppresses ferritinophagy, limits intracellular iron accumulation, and reduces ferroptotic sensitivity. Our findings establish a phosphorylation-dependent mechanism linking kinase signaling to ferritinophagy regulation and suggest Pin1 inhibition as a potential therapeutic strategy for ATC.

## Results

### Pin1 promotes ATC cell growth

Pin1 is a peptidyl-prolyl cis–trans isomerase that regulates numerous signaling proteins and plays essential roles in diverse biological processes, including tumorigenesis. Pin1 protein levels were markedly elevated in ATC compared with PTC or normal thyroid cells and tissues (Fig. S1A, B). The IHC staining confirmed stronger Pin1 expression in ATC tissues (Fig. S1C, D). Higher Pin1 expression was associated with poorer prognosis in ATC patients (Fig. S1E). To explore the biological role of Pin1 in ATC, stable knockdown was established in ATC cells, which significantly reduced Pin1 protein levels. Cell viability and colony formation assays showed that Pin1 knockdown significantly inhibited ATC cell growth (Fig. S1F–J). Consistently, Pin1 knockdown markedly suppressed tumor growth, as indicated by reduced tumor volume and weight (Fig. S1K–M). Collectively, these findings support that Pin1 promotes ATC progression both in vitro and in vivo.

### Knockdown Pin1 triggers ferroptosis in ATC cells

**Fig. 1:**
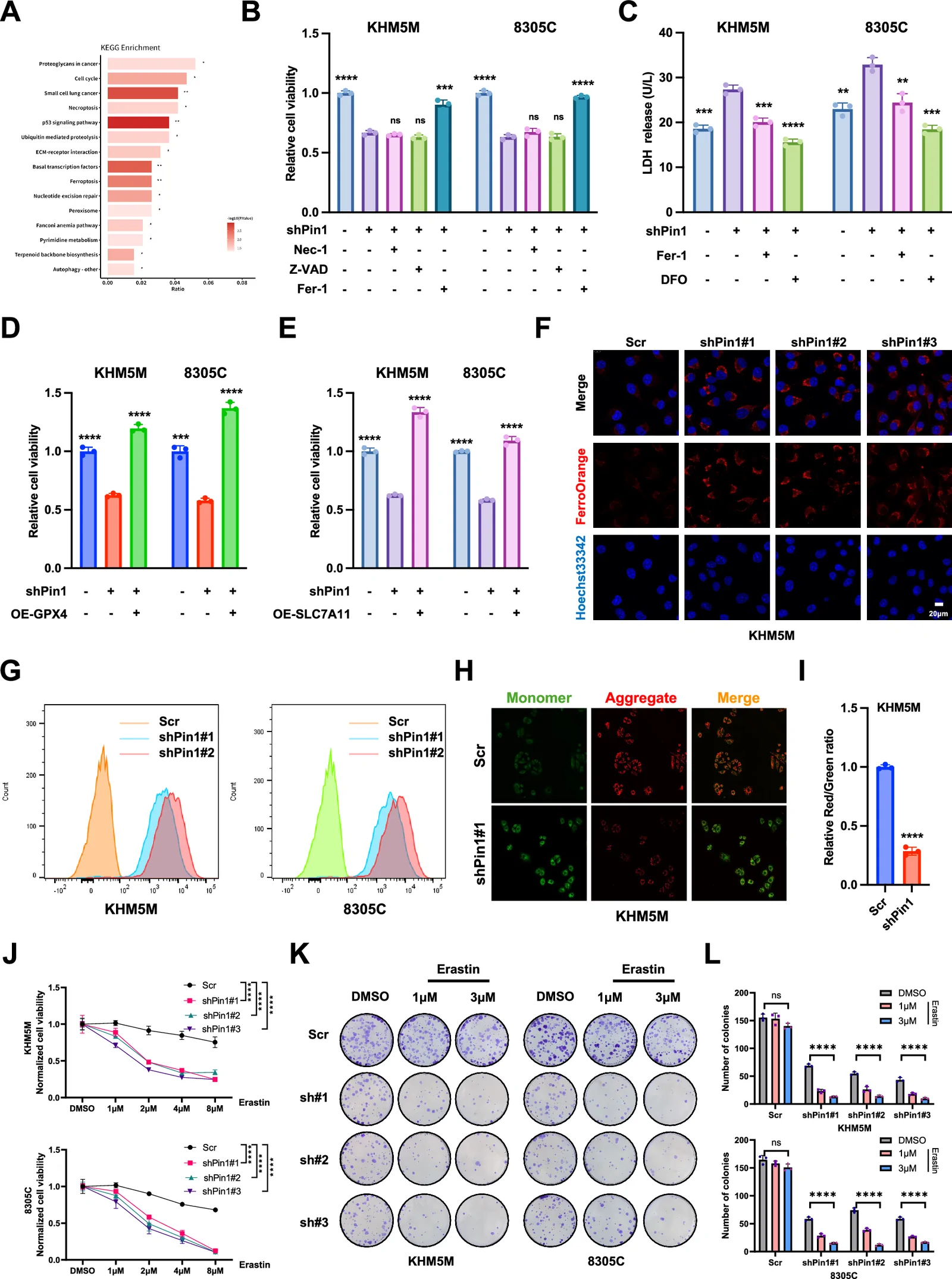
Pin1 depletion induces ferroptosis in ATC cells. **A** KEGG pathway enrichment analysis of differentially expressed proteins in Pin1-depleted versus control KHM5M cells identified by quantitative proteomics (*n* = 3). **B** Cell viability of Pin1-depleted KHM5M and 8305 C cells treated with necroptosis inhibitor necrostatin-1 (Nec-1, 10 μM), apoptosis inhibitor Z-VAD-FMK (Z-VAD, 20 μM), or ferroptosis inhibitor ferrostatin-1 (Fer-1, 2 μM) for 48 h. Data are mean ± s.d. (*n* = 3). ***P < 0.001, ****P < 0.0001 by two-way ANOVA test. **C** LDH release from Pin1-depleted KHM5M and 8305 C cells treated with Fer-1 (2 μM) or deferoxamine (DFO, 100 μM) for 24 h. Data are mean ± s.d. (*n* = 3). **P < 0.01, ***P < 0.001, ****P < 0.0001 by one-way ANOVA test. **D** Rescue of cell viability by GPX4 overexpression in Pin1-depleted KHM5M and 8305 C cells. Data are mean ± s.d. (*n* = 3). ***P < 0.001, ****P < 0.0001 by one-way ANOVA test. **E** Rescue of cell viability by SLC7A11 overexpression in Pin1-depleted KHM5M and 8305 C cells. Data are mean ± s.d. (*n* = 3). ****P < 0.0001 by one-way ANOVA test. **F** Representative confocal images of intracellular Fe²⁺ detected by FerroOrange staining in control and Pin1-depleted KHM5M cells. Nuclei were counterstained with Hoechst 33342. Scale bar, 20 μm. **G** Flow cytometric analysis of lipid peroxidation in Pin1-depleted KHM5M and 8305 C cells using C11-BODIPY 581/591 probe. **H** Mitochondrial membrane potential (ΔΨm) assessed by JC-1 staining in Pin1-depleted KHM5M cells. Representative confocal images showing JC-1 aggregates (red) and monomers (green). Scale bar, 50 μm. **I** Quantification of the relative JC-1 red/green fluorescence ratio in KHM5M cells shown in H. Data are mean ± s.d. (*n* = 3). ****P < 0.0001 by unpaired two-tailed Student’s *t* test. **J** Viability of Pin1-depleted KHM5M (top) and 8305 C (bottom) cells treated with erastin for 24 h, assessed by CCK-8 assay. Data are mean ± s.d. (*n* = 3). ****P < 0.0001 by two-way ANOVA test. **K** Representative images of colony formation assays using Pin1-depleted KHM5M (left) and 8305 C (right) cells treated with DMSO or erastin (1 μM or 3 μM) for 12 days. **L** Quantification of colony numbers from (**K**). Data are mean ± s.d. (*n* = 3). ^ns^P å 0.05, ****P < 0.0001 by two-way ANOVA test.

**Fig. 2:**
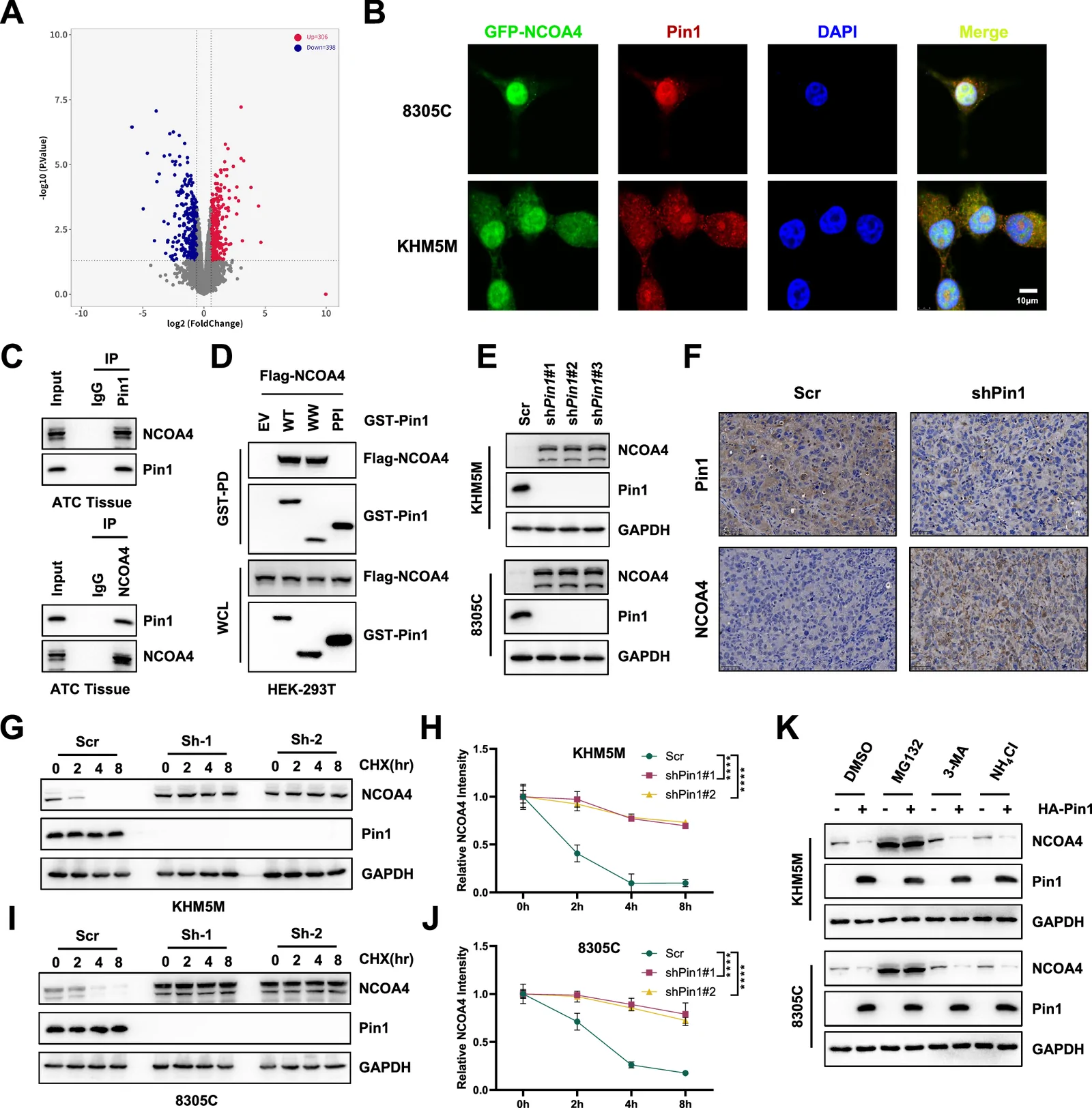
Pin1 interacts with and promotes proteasomal degradation of NCOA4. **A** Volcano plot showing differentially expressed proteins in Pin1-depleted versus control KHM5M cells analyzed by quantitative proteomics. Dashed lines indicate |logFC > 0.5| and adjusted *P* < 0.05 (*n* = 3). **B** Immunofluorescence staining of NCOA4 (green) and Pin1 (red) in KHM5M cells. Nuclei were counterstained with DAPI (blue). Merged images show colocalization. Scale bar, 10 μm. **C** Co-immunoprecipitation of endogenous Pin1 and NCOA4 from ATC patient tissues. Whole-cell lysates and immunoprecipitated (IP) samples were analyzed by immunoblotting. IgG served as negative control. **D** GST pull-down assay showing interaction between Flag-NCOA4 and GST-Pin1 domains in HEK-293T cells. Full-length, WW domain, and PPIase domain constructs were tested. GST alone served as negative control. **E** Immunoblot analysis of NCOA4 and Pin1 protein levels in control and Pin1-depleted KHM5M and 8305 C cells. **F** Representative immunohistochemical (IHC) staining of Pin1 and NCOA4 in xenograft tumors from control or Pin1-depleted KHM5M cells. Scale bar, 50 μm. Representative images from *n* = 7 mice per group. **G** Cycloheximide chase assay in Pin1-depleted KHM5M cells. Representative immunoblots showing NCOA4 protein levels at indicated time points after CHX treatment (100 μg/ml). **H** Quantification of NCOA4 protein half-life in G. Data are mean ± s.d. (*n* = 3). ****P < 0.0001 by two-way ANOVA test. **I** Cycloheximide chase assay in Pin1**-**depleted 8305 C cells. Representative immunoblots. **J** Quantification of NCOA4 half-life in (**I**). Data are mean ± s.d. (*n* = 3). ****P < 0.0001 by two-way ANOVA test. **K** Immunoblot analysis of NCOA4 in KHM5M and 8305 C cells treated with proteasome inhibitor MG132 (10 μM), lysosome inhibitor NH₄Cl (10 mM), or autophagy inhibitor 3-methyladenine (3-MA, 5 mM) for 12 h. DMSO served as vehicle control.

To investigate how Pin1 regulates ATC progression, we performed quantitative proteomic profiling of Pin1-knockdown and control KHM5M cells. Differentially expressed proteins were enriched in pathways related to the cell cycle, ferroptosis, and necroptosis (Fig. 1A). Growth inhibition caused by Pin1 silencing was rescued by the ferroptosis inhibitor ferrostatin-1 (Fer-1), but not by the necroptosis inhibitor Nec-1 or apoptosis inhibitor Z-VAD (Fig. 1B). Fer-1 treatment also significantly reduced LDH release in Pin1-deficient cells (Fig. 1C). To further strengthen the specificity of ferroptosis dependency, we ectopically expressed the canonical ferroptosis suppressors GPX4 and SLC7A11 in Pin1-deficient cells. Overexpression of either GPX4 or SLC7A11 significantly restored viability in both KHM5M and 8305 C cells, indicating that the viability loss caused by Pin1 depletion is predominantly ferroptosis-dependent (Fig. 1D, E). Pin1 knockdown markedly increased LDH release, malondialdehyde (MDA), hydrogen peroxide (H₂O₂), and intracellular ferrous iron levels (Fig. S2A–H). To further validate intracellular iron accumulation, FerroOrange staining was performed and demonstrated a marked increase in labile Fe²⁺ fluorescence intensity following Pin1 depletion (Fig. 1F). Consistent with this, lipid peroxidation was markedly increased in Pin1-silenced ATC cells (Fig. 1G). Loss of mitochondrial membrane potential is an early indicator of ferroptosis. JC-1 staining revealed that Pin1 depletion decreased the red-to-green fluorescence ratio, indicating mitochondrial membrane depolarization (Fig. 1H, I). Transmission electron microscopy revealed shrunken mitochondria with loss of cristae and ruptured outer membranes in Pin1-deficient cells—morphological hallmarks of ferroptosis (Fig. S2I). Pin1 depletion altered ferroptosis-related proteins, elevating NCOA4, LC3-II and ATG7 while suppressing SQSTM1, GPX4 and FTH1 (Figs. 2E and S2J). To further validate this role, we examined cell viability after treatment with the ferroptosis inducer erastin. Pin1 depletion significantly sensitized ATC cells to erastin-induced ferroptosis, as shown by reduced cell viability and colony formation (Fig. 1J–L). Together, these findings demonstrate that loss of Pin1 promotes ferroptosis in ATC cells.

### Pin1 facilitates NCOA4 degradation via K29/K48-linked ubiquitination

To identify the molecular mechanisms underlying Pin1-mediated ferroptosis, we screened the differentially expressed proteins and identified NCOA4 as a potential Pin1 substrate (Fig. 2A). NCOA4 promotes ferroptosis by mediating ferritinophagy, a selective autophagic process that degrades ferritin to release stored iron. Confocal microscopy revealed strong cytoplasmic co-localization of Pin1 and NCOA4 in KHM5M and 8305 C cells (Fig. 2B). Co-immunoprecipitation confirmed an endogenous interaction between Pin1 and NCOA4 in ATC tissues (Fig. 2C). Notably, endogenous co-immunoprecipitation assays further showed that treatment with the ferroptosis inducers erastin or RSL3 enhanced the interaction between Pin1 and NCOA4 in KHM5M cells, indicating that this association is dynamically strengthened under ferroptotic stress (Fig. S3A, B). Pin1 interacted with NCOA4 through its WW domain, the canonical motif responsible for substrate recognition (Fig. 2D).

Pin1 depletion increased NCOA4 protein abundance (Fig. 2E), whereas ectopic Pin1 expression—but not the catalytically inactive mutant (W34A/K63A)—reduced NCOA4 levels. This effect was reversed by the Pin1 inhibitor KPT-6566 (Fig. S3C, D). Immunohistochemical staining of xenograft tumors revealed an inverse correlation between Pin1 and NCOA4 expression (Fig. 2F). Pin1 overexpression shortened the NCOA4 half-life, whereas the inactive mutant had no effect (Fig. S3E–H). Conversely, Pin1 knockdown or pharmacologic inhibition enhanced NCOA4 protein stability (Figs. 2G–J and S3I–L).

**Fig. 3:**
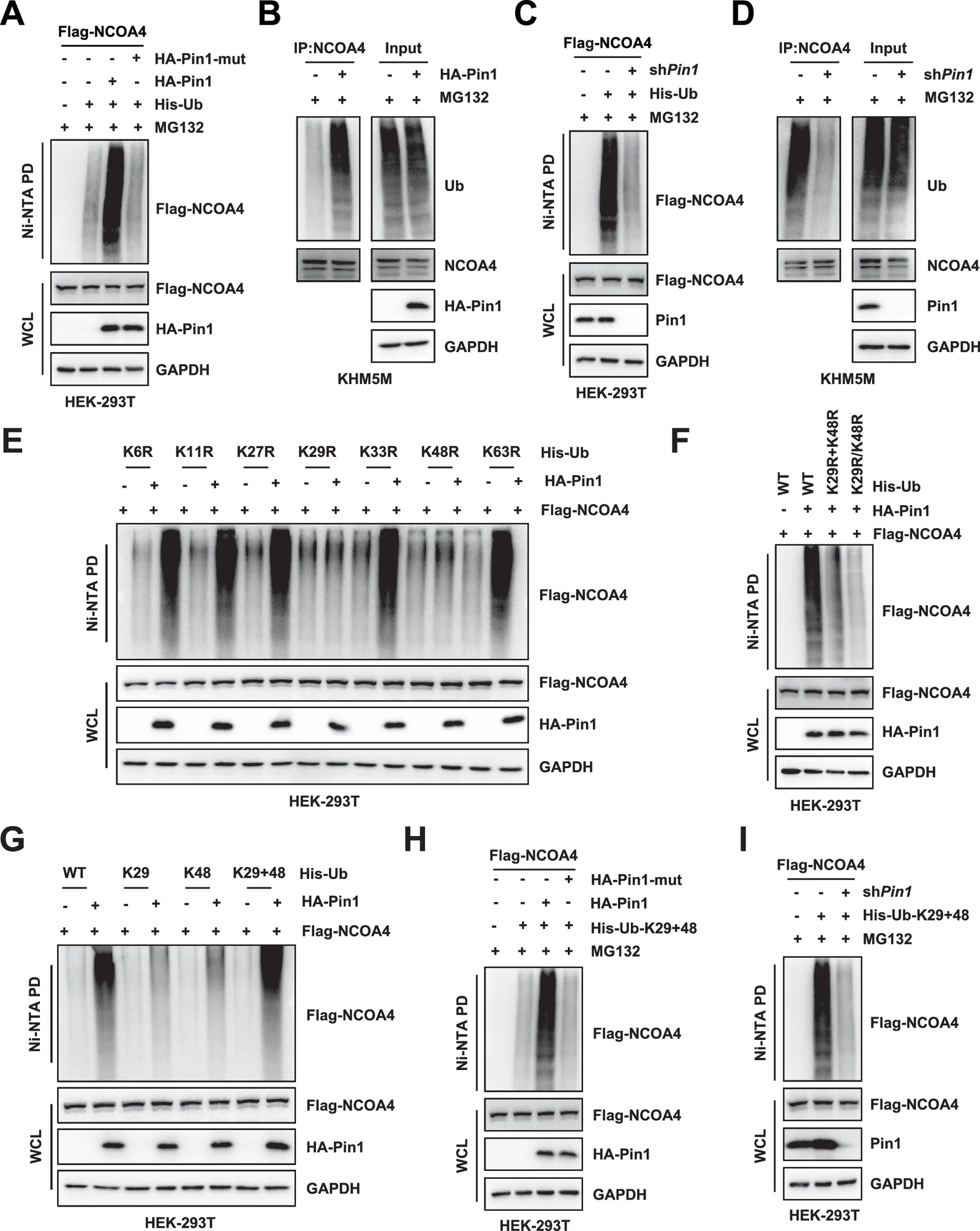
Pin1 promotes K29/K48-linked polyubiquitination of NCOA4. **A** In vivo ubiquitination assay in HEK-293T cells co-transfected with Flag-NCOA4, His-Ub, and HA-Pin1 wild-type (WT) or catalytically inactive Pin1 (W34A/K63A). Cells were treated with MG132 (10 μM) for 12 h before harvest. Ubiquitinated NCOA4 was enriched by Ni-NTA pull-down and detected by anti-Flag immunoblotting. **B** In vivo ubiquitination assay in KHM5M cells overexpressing HA-Pin1. Endogenous NCOA4 ubiquitination was detected after NCOA4 immunoprecipitation. **C** In vivo ubiquitination assay showing reduced NCOA4 ubiquitination in Pin1-depleted HEK-293T cells. **D** In vivo ubiquitination assay showing reduced endogenous NCOA4 ubiquitination in Pin1-depleted KHM5M cells. **E** In vivo ubiquitination assay using lysine-only ubiquitin mutants (His-Ub-K6, K11, K27, K29, K33, K48, K63-only) in HEK-293T cells co-expressing Flag-NCOA4 and HA-Pin1. **F** In vivo ubiquitination assay comparing K29R/K48R double mutant (K29/48R) versus co-transfection of K29R and K48R single mutants. **G** In vivo ubiquitination assay using K29-only or K48-only ubiquitin constructs. **H** Immunoblot analysis of K29- and K48-linked NCOA4 ubiquitination in Pin1-overexpressing HEK-293T cells. **I** Immunoblot analysis of K29- and K48-linked NCOA4 ubiquitination in Pin1-depleted HEK-293T cells. **A–I** All ubiquitination assays were performed in cells treated with MG132 (10 μM) for 12 h before harvest.

Treatment with the proteasome inhibitor MG132—but not the autophagy inhibitor 3-methyladenine (3-MA) or lysosome inhibitor NH₄Cl—blocked Pin1-mediated NCOA4 degradation (Fig. 2K), indicating a proteasome-dependent pathway. Pin1 overexpression increased both exogenous and endogenous NCOA4 ubiquitination (Fig. 3A, B). In contrast, the inactive Pin1 mutant or Pin1 depletion failed to enhance NCOA4 ubiquitination (Fig. 3C, D). To identify the ubiquitin linkage involved, we performed in vivo ubiquitination assays using a panel of ubiquitin mutants and found that K29- and K48-linked chains most strongly supported Pin1-induced NCOA4 ubiquitination (Fig. 3E). The K29/K48R double mutant markedly impaired Pin1-induced NCOA4 ubiquitination, unlike single K29R or K48R mutants (Fig. 3F). Both K29-only and K48-only ubiquitin constructs partially restored this effect (Fig. 3G). Consistent with these results, Pin1 enhanced K29- and K48-linked polyubiquitination of NCOA4, whereas Pin1 inactivation or depletion abolished this effect (Fig. 3H, I). Together, these findings demonstrate that Pin1 promotes NCOA4 degradation through K29/K48-linked ubiquitination.

### Pin1 regulates ferroptosis through NCOA4-mediated ferritinophagy

Given that Pin1 promotes the proteasomal degradation of NCOA4, we reasoned that the ferroptosis-inducing effect of Pin1 depletion might depend on NCOA4. To test this, we performed NCOA4 knockdown in Pin1-deficient ATC cells. Functionally, NCOA4 depletion markedly suppressed ferroptotic features in Pin1-deficient cells. Levels of LDH release, MDA, intracellular H₂O₂, and Fe²⁺ accumulation were significantly decreased when NCOA4 was silenced (Fig. S4A–H). Lipid peroxidation, a hallmark of ferroptosis, was also markedly reduced, and JC-1 staining revealed restoration of mitochondrial membrane potential (Fig. S4I–L). NCOA4 silencing reversed the ferritinophagy-associated changes induced by Pin1 depletion, as evidenced by restoration of FTH1 and GPX4 expression, reduction of ATG7 and LC3-II accumulation, and normalization of SQSTM1 levels, while effectively reducing NCOA4 expression without affecting Pin1 depletion efficiency (Fig. S4M). These findings further support that the ferroptotic phenotype induced by Pin1 loss is largely dependent on NCOA4-mediated ferritinophagy.

Importantly, NCOA4 knockdown abolished the increased sensitivity of Pin1-deficient ATC cells to the ferroptosis inducer erastin, indicating that NCOA4 is required for Pin1-mediated ferroptosis regulation (Fig. S5A–C). To further determine whether this effect depends on autophagy/ferritinophagy, we treated Pin1-deficient ATC cells with the autophagy inhibitor chloroquine (CQ). CQ markedly attenuated the increased sensitivity of shPin1 cells to erastin, as demonstrated by restored cell viability and colony formation in both KHM5M and 8305 C cells (Fig. S5D–F). Collectively, these findings demonstrate that Pin1 regulates ferroptosis through NCOA4-dependent ferritinophagy, and that loss of NCOA4 can rescue the ferroptotic phenotype caused by Pin1 depletion.

### The phosphorylation of NCOA4 at Ser572 mediated by CDK2 promotes its ubiquitination

**Fig. 4:**
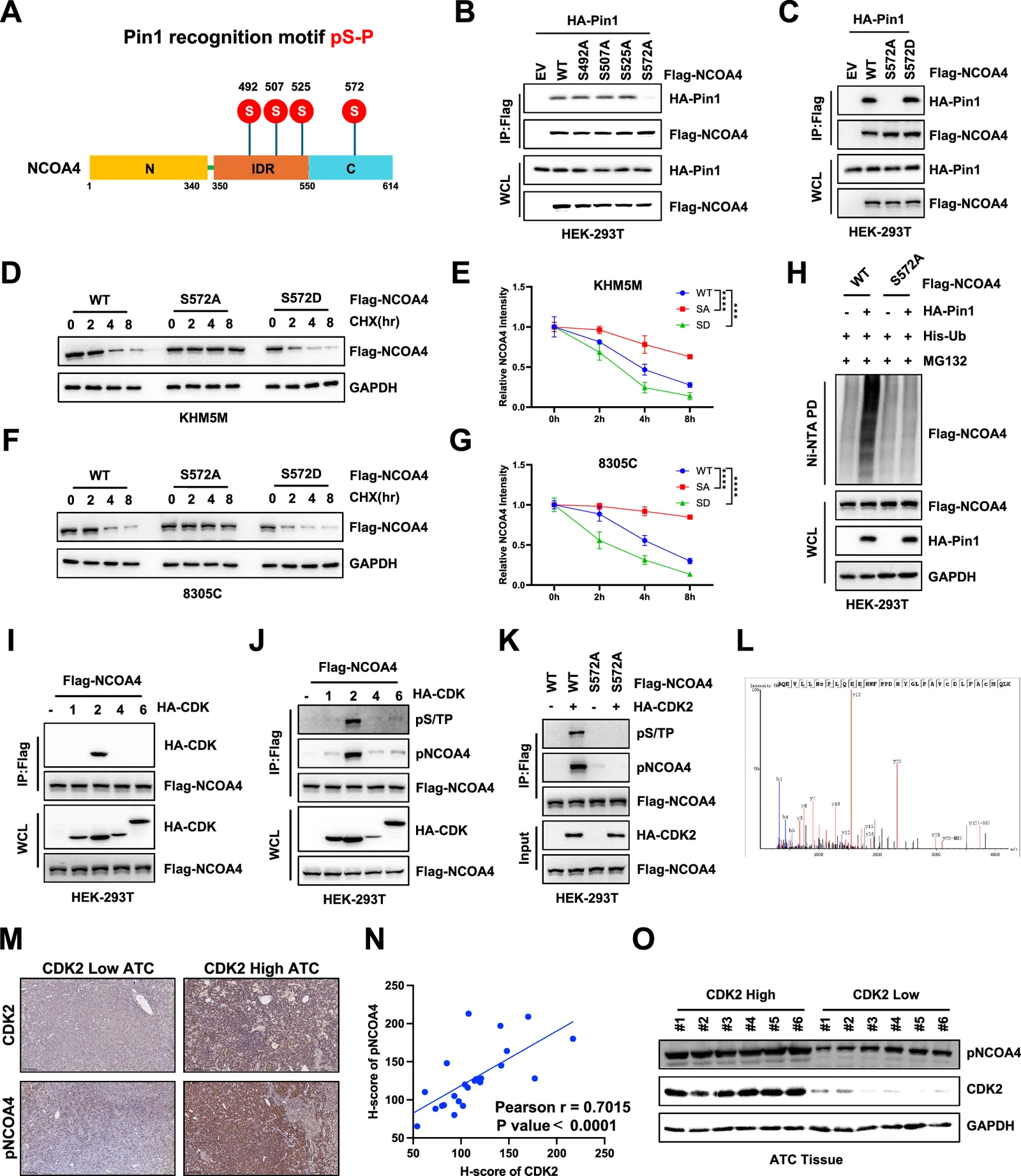
CDK2 phosphorylates NCOA4 at Ser572 to facilitate Pin1 binding. **A** Schematic representation of human NCOA4 protein showing potential CDK phosphorylation sites (Ser/Thr-Pro motifs). The four candidate sites (S492, S507, S525, S572) are indicated. **B** Flag-immunoprecipitation assay showing interaction between HA-Pin1 and Flag-NCOA4 SA mutants in HEK-293T cells. Only S572A abolished Pin1 binding. **C** Flag-immunoprecipitation assay comparing Pin1 binding to NCOA4 WT, phospho-deficient (S572A), and phospho-mimetic (S572D) mutants. Representative of three independent experiments. **D** Cycloheximide chase assay in KHM5M cells expressing NCOA4-WT, S572A, or S572D. Representative immunoblots showing NCOA4 protein levels at indicated time points after CHX treatment (100 μg/ml). **E** Quantification of NCOA4 protein half-life in (**D**). Data are mean ± s.d. (*n* = 3). ***P < 0.001, ****P < 0.0001 by two-way ANOVA test. **F** Cycloheximide chase assay in 8305 C cells expressing NCOA4 variants. Representative immunoblots showing NCOA4 protein levels at indicated time points after CHX treatment (100 μg/ml). **G** Quantification of NCOA4 protein half-life in (**F**). Data are mean ± s.d. (*n* = 3). ****P < 0.0001 by two-way ANOVA test. **H** In vivo ubiquitination assay showing that the S572A mutation reduces Pin1-mediated NCOA4 ubiquitination. HEK-293T cells were co-transfected with Flag-NCOA4-WT or Flag-NCOA4-S572A, His-Ub, and HA-Pin1, treated with MG132 (10 μM) for 12 h before harvest. **I** Flag-immunoprecipitation assay screening CDK family kinases (CDK1, CDK2, CDK4, CDK6) for interaction with NCOA4. **J** Immunoblot analysis of WCL and Flag-immunoprecipitation derived from HEK-293T cells transfected with CDK family kinases and Flag-NCOA4. **K** Immunoblot analysis of WCL and Flag-immunoprecipitation products derived from HEK-293T cells transfected with indicated constructed. **L** LC-MS/MS analysis of Flag-immunoprecipitated NCOA4 from HEK-293T cells co-expressing HA-CDK2. **M** Representative immunohistochemical images of CDK2 and pNCOA4(S572) staining in CDK2-low and CDK2-high ATC tissues. **N** Correlation analysis between CDK2 H-score and pNCOA4 H-score in ATC tissues. Pearson r = 0.7015, *P* < 0.0001. **O** Immunoblot analysis of pNCOA4(S572) and CDK2 in representative CDK2-high and CDK2-low ATC tissue samples.

We previously reported that Pin1 regulates the stability of phosphorylated proteins by selectively recognizing Ser/Thr–Pro motifs [24]. We focused on four canonical Pin1-recognition motifs in NCOA4—Ser492–Pro493, Ser507–Pro508, Ser525–Pro526, and Ser572–Pro573 (Fig. 4A). Among these sites, the S572A mutation markedly reduced Pin1 binding, whereas the phosphomimetic S572D mutation slightly enhanced the interaction, indicating S572 phosphorylation–dependent recognition by Pin1 (Fig. 4B–C). The non-phosphorylatable S572A mutant resisted degradation, as evidenced by a prolonged NCOA4 half-life (Fig. 4D–G). Consistently, the S572A mutant abolished Pin1-mediated NCOA4 ubiquitination (Fig. 4H). Together, these findings indicate that Pin1 promotes NCOA4 ubiquitination in an S572 phosphorylation–dependent manner.

Cyclin-dependent kinases (CDKs) phosphorylate Ser/Thr–Pro motifs and often cooperate with Pin1 to modulate substrate conformation and stability [25]. To identify the kinase responsible for NCOA4-S572 phosphorylation, we examined interactions between NCOA4 and a panel of CDKs. Only CDK2 specifically interacted with NCOA4 under both ectopic and physiological conditions (Figs. 4I and S6A). Bioinformatic analysis identified a conserved serine–proline (SP) motif at S572 that matches the canonical CDK2 consensus (pS/T–P) (Fig. S6B). A CDK substrate antibody detected CDK2-dependent phosphorylation of NCOA4 (Fig. 4J), and the non-phosphorylatable S572A mutant abolished this signal (Fig. 4K). We generated and validated a phosphorylation-specific antibody recognizing NCOA4-pS572 (Fig. S6C). Consistent with these results, mass spectrometry confirmed CDK2-dependent phosphorylation of NCOA4 at Ser572 (Fig. 4L). Pharmacologic inhibition of CDK2 (CVT-313) or CDK2 depletion markedly reduced NCOA4-pS572 levels (Fig. S6D, E). To extend these findings to clinical samples, we examined CDK2 and pNCOA4(S572) expression in ATC tissues. The IHC staining showed that CDK2-high ATC samples exhibited stronger pNCOA4(S572) staining than CDK2-low ATC samples (Fig. 4M). Consistently, correlation analysis revealed a significant positive association between CDK2 H-score and pNCOA4 H-score in ATC tissues (Pearson r = 0.7015, *P* < 0.0001; Fig. 4N). Immunoblot analysis of representative ATC tissue lysates further confirmed that CDK2-high samples tended to express higher levels of pNCOA4(S572) than CDK2-low samples (Fig. 4O). Collectively, these findings establish CDK2 as the upstream kinase responsible for NCOA4 Ser572 phosphorylation in ATC.

We next asked whether CDK2-mediated phosphorylation of NCOA4 affects its degradation. CDK2 depletion or inhibition attenuated Pin1–NCOA4 interaction (Fig. S6F, G). Accordingly, CDK2 depletion or inhibition increased NCOA4 protein abundance and prolonged its half-life in ATC cells (Fig. S6H–M). Moreover, CDK2 inhibition suppressed Pin1-mediated NCOA4 ubiquitination (Fig. S6N). Together, these observations demonstrate that CDK2-mediated phosphorylation of NCOA4 at Ser572 enables Pin1 binding, thereby promoting NCOA4 ubiquitination and proteasomal degradation.

### Phosphorylation of NCOA4 at Ser572 suppresses ferritinophagy

**Fig. 5:**
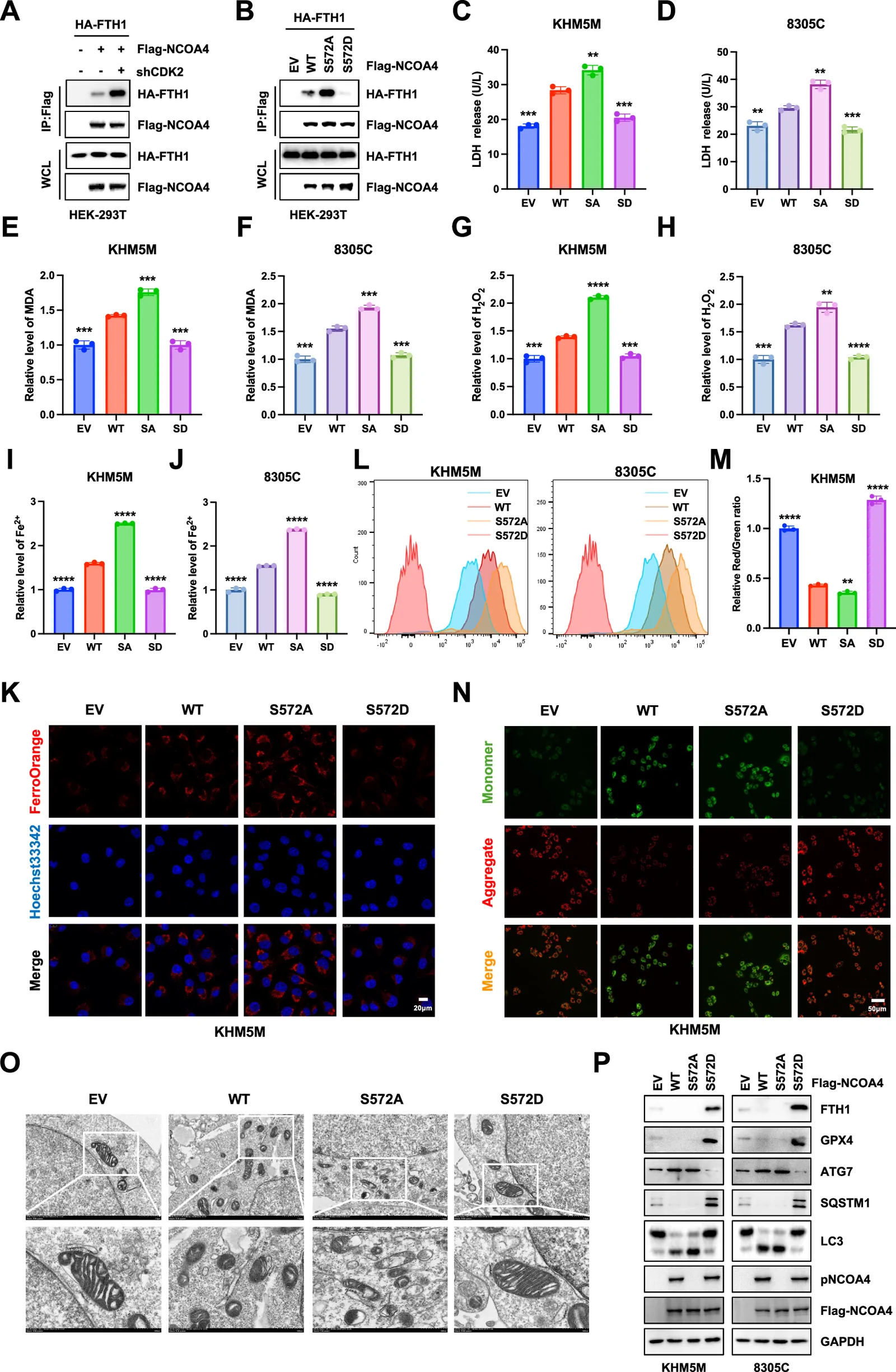
Phosphorylation of NCOA4 at Ser572 inhibits ferritinophagy and ferroptosis. **A** Flag-immunoprecipitation assay showing the interaction between Flag-NCOA4 and HA-FTH1 in HEK-293T cells with or without CDK2 knockdown. Cell lysates were immunoprecipitated with anti-Flag antibody and analyzed by immunoblotting with the indicated antibodies. **B** Flag-immunoprecipitation assay showing the interaction between HA-FTH1 and Flag-tagged NCOA4 variants (WT, phospho-deficient mutant S572A, and phospho-mimetic mutant S572D) in HEK-293T cells. Cell lysates were immunoprecipitated with anti-Flag antibody and analyzed by immunoblotting with the indicated antibodies. LDH release from KHM5M (**C**) and 8305 C (**D**) cells stably expressing NCOA4-WT, phospho-deficient mutant (S572A), or phospho-mimetic mutant (S572D). Data are mean ± s.d. (*n* = 3). **P < 0.01, ***P < 0.001 by one-way ANOVA test. Intracellular malondialdehyde (MDA) levels in KHM5M (**E**) and 8305 C (**F**) cells expressing NCOA4 variants. Data are mean ± s.d. (*n* = 3). ***P < 0.001 by one-way ANOVA test. Intracellular hydrogen peroxide (H₂O₂) levels in KHM5M (**G**) and 8305 C (**H**) cells expressing NCOA4 variants. Data are mean ± s.d. (*n* = 3). **P < 0.01, ***P < 0.001, ****P < 0.0001 by one-way ANOVA test. Intracellular ferrous iron (Fe²⁺) levels in KHM5M (**I**) and 8305 C (**J**) cells expressing NCOA4 variants measured by colorimetric assay. Data are mean ± s.d. (*n* = 3). ****P < 0.0001 by one-way ANOVA test. **K** Representative confocal images of intracellular Fe²⁺ detected by FerroOrange staining in KHM5M cells expressing EV, NCOA4-WT, S572A, or S572D. Nuclei were counterstained with Hoechst 33342. Scale bar, 20 μm. **L** Flow cytometric analysis of lipid peroxidation in KHM5M and 8305 C cells expressing NCOA4 variants using C11-BODIPY 581/591 probe. **M** Quantification of the relative JC-1 red/green fluorescence ratio in KHM5M cells expressing EV, NCOA4-WT, S572A, or S572D, corresponding to the representative images shown in (**N**). Data are mean ± s.d. (*n* = 3). **P < 0.01, ****P < 0.0001 by one-way ANOVA test. **N** Mitochondrial membrane potential (ΔΨm) assessed by JC-1 staining in KHM5M cells expressing NCOA4 variants. Representative confocal images showing JC-1 aggregates (red) and monomers (green). Scale bar, 50 μm. **O** Transmission electron microscopy images showing mitochondrial morphology in KHM5M cells expressing NCOA4 variants. White boxes highlight mitochondria. NCOA4-WT and S572A induced loss of cristae and outer membrane rupture, whereas S572D maintained normal mitochondrial structure. Scale bars, 1 μm (upper panels), 0.5 μm (lower panels). **P** Immunoblot analysis of ferritinophagy-related markers (NCOA4, FTH1, ATG7, LC3, SQSTM1, GPX4) in KHM5M and 8305 C cells expressing NCOA4 variants.

Given that NCOA4-mediated ferritinophagy depends on ferritin binding, we first examined the interaction between NCOA4 and FTH1. Flag-immunoprecipitation assays showed that CDK2 knockdown increased NCOA4–FTH1 binding (Fig. 5A). Consistently, relative to WT NCOA4, the S572A mutant exhibited stronger association with FTH1, whereas the S572D mutant displayed markedly reduced binding (Fig. 5B), suggesting that Ser572 phosphorylation weakens the ferritin-binding capacity of NCOA4. Functionally, S572A promoted, whereas S572D suppressed, ferroptotic phenotypes, as reflected by corresponding changes in LDH release, MDA, intracellular H₂O₂, and Fe²⁺ levels (Fig. 5C–J). FerroOrange staining further confirmed that WT and especially S572A increased intracellular labile Fe²⁺ accumulation, whereas S572D reduced intracellular Fe²⁺ levels (Fig. 5K). Consistent with these findings, S572A enhanced lipid peroxidation, while S572D reduced it (Fig. 5L). JC-1 staining showed that WT and especially S572A reduced the JC-1 red-to-green fluorescence ratio, whereas S572D maintained a higher red/green ratio, indicating that S572 phosphorylation attenuates mitochondrial depolarization (Fig. 5M, N). Moreover, transmission electron microscopy revealed that WT or S572A overexpression induced shrunken mitochondria with loss of cristae and outer-membrane rupture, whereas S572D largely preserved mitochondrial morphology (Fig. 5O). At the molecular level, WT and S572A increased LC3-II and ATG7 while reducing FTH1, whereas S572D showed the opposite pattern; similar reciprocal changes were observed in GPX4, SQSTM1, and pNCOA4 (Fig. 5P). Together, these results indicate that phosphorylation of NCOA4 at Ser572 suppresses ferritin binding, ferritinophagy, and ferroptosis.

To further investigate the link between NCOA4 Ser572 phosphorylation and ferritinophagy, we compared the erastin sensitivity of ATC cells overexpressing WT, S572A, or S572D. WT and S572A increased erastin sensitivity, whereas S572D decreased it (Fig. S7A–C). Consistently, WT and S572A significantly suppressed tumor growth (reduced tumor volume and weight), whereas S572D attenuated the tumor-suppressive effect of NCOA4 (Fig. S7D–F). IHC assays of mouse tumors revealed that NCOA4-S572D increased FTH and Ki-67 levels (Fig. S7G). These data further support that phosphorylation of NCOA4 at Ser572 inhibits ferritinophagy, limits intracellular iron release, suppresses ferroptosis, and thereby promotes ATC progression.

### Targeting Pin1 suppresses ATC tumor growth by promoting ferroptosis

**Fig. 6:**
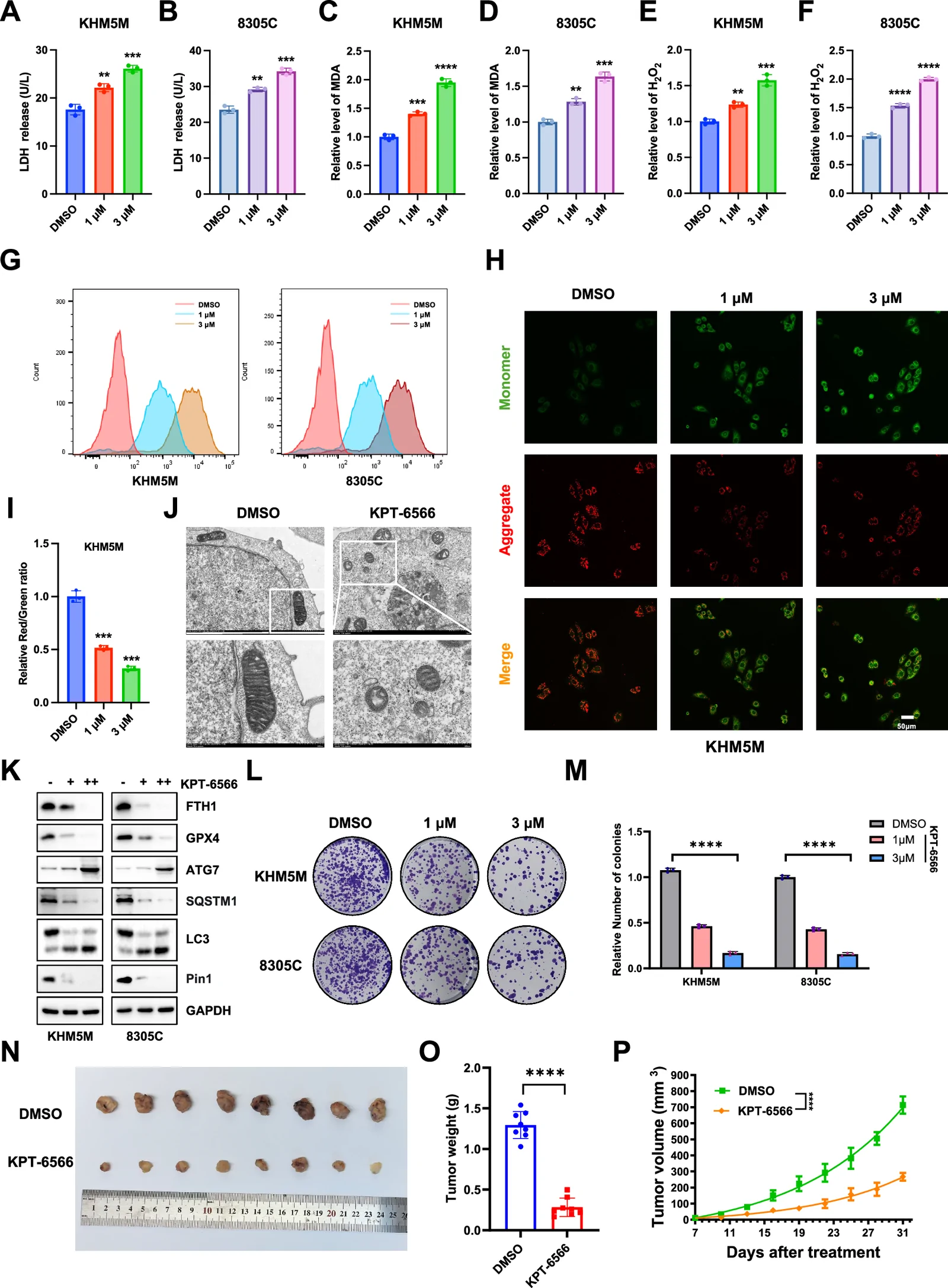
Pin1 inhibitor KPT-6566 induces ferroptosis and suppresses ATC tumor growth. LDH release from KHM5M (**A**) and 8305 C (**B**) cells treated with increasing concentrations of KPT-6566 for 24 h. Data are mean ± s.d. (*n* = 3). **P < 0.01, ***P < 0.001 by one-way ANOVA test. Intracellular MDA levels in KHM5M (**C**) and 8305 C (**D**) cells treated with indicated concentrations of KPT-6566 for 24 h. Data are mean ± s.d. (*n* = 3). **P < 0.01, ***P < 0.001, ****P < 0.0001 by one-way ANOVA test. Intracellular H₂O₂ levels in KHM5M (**E**) and 8305 C (**F**) cells treated with indicated concentrations of KPT-6566 for 24 h. Data are mean ± s.d. (*n* = 3). **P < 0.01, ***P < 0.001, ****P < 0.0001 by one-way ANOVA test. **G** Flow cytometric analysis of lipid peroxidation in KHM5M and 8305 C cells treated with indicated concentrations of KPT-6566 for 24 h using C11-BODIPY 581/591 probe. **H** Mitochondrial membrane potential (ΔΨm) assessed by JC-1 staining in KHM5M cells treated with indicated concentrations of KPT-6566 for 24 h. Representative confocal images showing JC-1 aggregates (red) and monomers (green). Scale bar, 50 μm. **I** Quantification of the relative JC-1 red/green fluorescence ratio in KHM5M cells treated with DMSO or KPT-6566 (1 μM or 3 μM), corresponding to the representative images shown in H. Data are mean ± s.d. (*n* = 3). ***P < 0.001 by one-way ANOVA test. **J** Transmission electron microscopy images showing mitochondrial morphology in KHM5M cells treated with DMSO or KPT-6566 (1 μM) for 24 h. White boxes highlight mitochondria. KPT-6566 treatment induced loss of cristae and outer membrane rupture. Scale bars, 1 μm (upper panels), 0.5 μm (lower panels). **K** Immunoblot analysis of ferritinophagy-related markers (NCOA4, FTH1, ATG7, LC3, SQSTM1, GPX4) in **K**HM5M and 8305 C cells treated with KPT-6566 (1 μM or 3 μM) for 24 h. **L** Representative images of colony formation assays using KHM5M and 8305 C cells treated with indicated concentrations of KPT-6566 for 12 days. **M** Quantification of colony numbers from (**L**). Data are mean ± s.d. (*n* = 3). ****P < 0.0001 by one-way ANOVA test. Xenograft tumor growth in nude mice injected with KHM5M cells and treated with vehicle or KPT-6566 (20 mg/kg**)** three times per week for 21 days. Representative images of dissected tumors (**N**), tumor weights (**O**), and tumor growth curves (**P**). Data are mean ± s.d. (*n* = 8 mice per group). ****P < 0.0001 by unpaired t-test (**O**) or two-way ANOVA test (**P**).

Given the role of Pin1 in ferroptosis regulation, we reasoned that pharmacological inhibition of Pin1 might enhance ferroptosis and thereby suppress ATC progression. Consistent with this hypothesis, treatment with the Pin1 inhibitor KPT-6566 increased LDH release, MDA content, intracellular H₂O₂, and Fe²⁺ levels in ATC cells (Fig. 6A–F). KPT-6566 also enhanced lipid peroxidation and disrupted mitochondrial membrane potential, as evidenced by a decreased JC-1 red-to-green ratio and ultrastructural mitochondrial damage (Fig. 6G–I). In parallel, KPT-6566 induced ferritinophagy-associated molecular changes, including increased NCOA4, ATG7, and LC3-II and decreased FTH1, GPX4, and SQSTM1, indicating activation of ferritinophagy and ferroptosis (Fig. 6J, K).

Based on these findings, we next evaluated the antitumor effect of KPT-6566 in vivo. As expected, KPT-6566 treatment significantly suppressed xenograft growth, as reflected by reduced colony formation in vitro and decreased tumor volume and tumor weight in vivo (Fig. 6L–P). To further determine whether this antitumor effect was accompanied by ferroptosis-associated changes in tumors, we analyzed xenograft tissues by immunoblotting and immunohistochemistry. KPT-6566-treated tumors showed increased NCOA4 expression, reduced FTH1 levels, and decreased Ki67 staining, consistent with enhanced ferritinophagy/ferroptosis and reduced proliferative activity in vivo (Fig. S8A, B). Moreover, H&E staining of major organs, including the heart, liver, spleen, lung, and kidney, revealed no obvious histopathological abnormalities in KPT-6566-treated mice, supporting acceptable systemic tolerability under the treatment conditions used in this study (Fig. S8C). Together, these results indicate that pharmacologic inhibition of Pin1 suppresses ATC tumor growth in association with activation of the NCOA4–ferritinophagy–ferroptosis axis, while exhibiting minimal systemic toxicity in vivo.

## Discussion

**Fig. 7:**
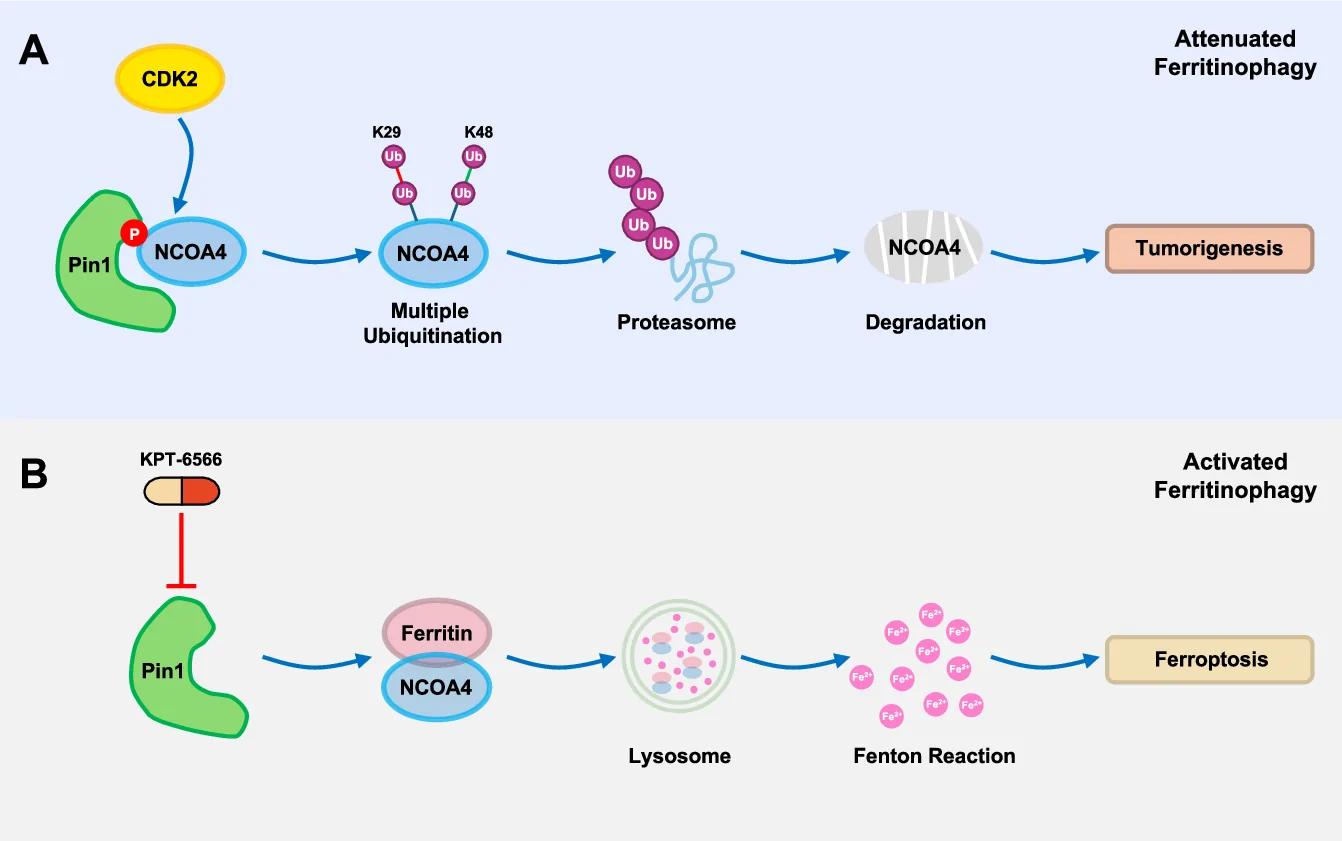
Proposed model of Pin1-mediated regulation of ferroptosis in ATC. **A** In ATC cells, CDK2 phosphorylates NCOA4 at Ser572, creating a binding site for Pin1. Pin1 recognizes phosphorylated NCOA4 via its WW domain and promotes K29/K48-linked ubiquitination and subsequent proteasomal degradation of NCOA4. This suppresses NCOA4-mediated ferritinophagy, reduces intracellular iron availability and lipid peroxidation, thereby inhibiting ferroptosis and promoting tumor growth. **B** Pin1 inhibitor KPT-6566 prevents NCOA4 degradation, restoring ferritinophagy activity. Enhanced ferritinophagy increases intracellular ferrous iron levels and lipid peroxidation, inducing ferroptosis and suppressing ATC tumor growth. This highlights Pin1 inhibition as a promising therapeutic strategy for ATC treatment.

Ferroptosis is an iron-dependent form of programmed cell death characterized by the accumulation of lipid peroxides [29, 30]. However, the molecular mechanisms governing ferroptosis regulation in ATC remain poorly defined. In this study, we identify Pin1 as a key negative regulator of ferroptosis that ultimately promotes ATC progression. Mechanistically, Pin1 binds to phosphorylated NCOA4 in a CDK2-dependent manner, facilitating K29/K48-linked ubiquitination and subsequent proteasomal degradation of NCOA4. This process suppresses ferritinophagy-mediated iron mobilization, thereby limiting intracellular iron availability and lipid peroxidation. Conversely, pharmacological inhibition of Pin1 enhances ferritinophagy-mediated iron mobilization, increases lipid peroxide accumulation, and restrains tumor growth. Together, these findings define a previously unrecognized CDK2–Pin1–NCOA4 regulatory axis that links phosphorylation-dependent protein turnover to ferroptosis control in ATC (Fig. 7).

ATC is a rare and highly malignant thyroid tumor with dismal prognosis and few effective treatment options [3, 31, 32]. Because of its dedifferentiated phenotype, ATC exhibits aggressive invasion, rapid progression, and therapeutic resistance [2, 4]. Thus, elucidating new molecular mechanisms driving ATC progression is crucial for developing novel therapeutic strategies. Pin1, the only known peptidyl-prolyl cis–trans isomerase (PPIase), regulates the conformational state of phosphorylated Ser/Thr–Pro motifs [22, 23]. Pin1 is highly expressed in multiple cancers and correlates with poor patient prognosis [26, 28, 33]. Pin1 regulates oncogenes and tumor suppressors, mediating critical crosstalk among oncogenic pathways that control cell cycle, proliferation, motility, apoptosis, and immune evasion [24, 26–28]. Consistent with these findings, our data show that Pin1 is highly expressed in ATC and correlates with poor prognosis. Functionally, Pin1 knockdown markedly suppressed ATC tumor growth both in vitro and in vivo. Importantly, our study expands the oncogenic role of Pin1 by demonstrating that its tumor-promoting activity is mediated, at least in part, through suppression of ferroptosis.

Pin1 functions as a critical hub that senses and coordinates phosphorylation-dependent signaling networks [23, 34]. Structurally, Pin1 contains an N-terminal WW domain that recognizes phospho-Ser/Thr–Pro motifs and a C-terminal PPIase domain that catalyzes cis–trans isomerization of target substrates [35, 36]. Here, we identified NCOA4 as a new substrate of Pin1 that specifically binds to its WW domain. The interaction between Pin1 and its substrates is phosphorylation dependent. Proline-directed kinases, including CDKs, are involved in this process [27, 37]. It has been reported that CDK1 directly phosphorylated pVHL at Ser80, thereby promoting its binding to Pin1 [25]. Our study revealed that CDK2 phosphorylates NCOA4 to facilitate its interaction with Pin1. Specifically, CDK2-mediated phosphorylation at Ser572 enables NCOA4–Pin1 binding. Pin1-induced conformational changes trigger subsequent post-translational modifications of its substrates, altering their localization, stability, and activity [24, 25, 38–41]. Recent studies have shown that Pin1 is associated with the ubiquitination and degradation of multiple tumor suppressors [24, 25]. For example, Pin1 destabilizes FBXW7 by disrupting its dimerization and promoting self-ubiquitination, thereby relieving oncogene suppression [41]. Similarly, Pin1 promotes VHL degradation, ultimately driving triple-negative breast cancer progression [25]. Our previous research showed that Pin1 facilitates SIK1 interaction with the E3 ligase ITCH to promote its ubiquitination and degradation [24]. In this study, we demonstrate that Pin1 promotes the K29/K48-linked ubiquitination process and proteasomal degradation of NCOA4.

Ferroptosis is a regulated cell death pathway driven by iron accumulation and lipid peroxidation [5–7]. Its execution relies on dysregulated iron metabolism and redox imbalance [8]. Aberrant ferroptosis regulation is tightly linked to tumor progression [9–12]. Cancer cells often develop mechanisms to evade ferroptosis, thereby sustaining growth and metastasis [42–45]. Genes that promote ferroptosis, such as DPP4, ISCU, TIMP1, and BID, correlate with better prognosis in thyroid cancer, whereas inhibitors such as GPX4 and GSS associate with poor outcomes [11, 46, 47]. Ferroptosis contributes to the initiation and progression of thyroid cancer. GPX4, which maintains redox homeostasis, is highly expressed in thyroid cancer, reduces ROS production, increases glutathione levels, and thereby inhibits ferroptosis and promotes tumor growth [46, 48–50]. Our data demonstrate that Pin1 inhibits ferroptosis in ATC by suppressing NCOA4-mediated ferritinophagy. Importantly, rescue experiments using GPX4 and SLC7A11 overexpression further confirm that the cytotoxic effect induced by Pin1 depletion is predominantly ferroptosis-dependent rather than nonspecific cell death. Ferritinophagy is a selective form of autophagy targeting ferritin for degradation, thereby releasing iron and inducing ferroptosis [13]. This process is mediated by NCOA4, which binds ferritin and directs it to autophagosomes for lysosomal degradation, releasing ferrous iron [14–16]. Ferritinophagy shares features with general autophagy, including altered ATG5, ATG7, and SQSTM1 expression [13, 14].

Post-translational modifications of NCOA4 profoundly influence ferritinophagy and ferroptosis, thereby affecting tumor initiation and progression. ATM-mediated phosphorylation stabilizes NCOA4, enhances ferritin binding, and promotes ferritinophagy and ferroptosis [21]. Protein ubiquitination is a dynamic post-translational modification initiated by covalent attachment of ubiquitin to a lysine residue on target proteins [51, 52]. Ubiquitin can form chains through one of its seven lysine residues (K6, K11, K27, K29, K33, K48, K63), generating polyubiquitin linkages with distinct functional outcomes [53, 54]. K48-linked chains are the most common and typically target proteins for proteasomal degradation [54]. Ubiquitination of NCOA4 directly determines its abundance and stability. E3 ligases TRIM7, HERC2, and DTX2 mediate K48-linked ubiquitination of NCOA4, thereby impairing ferritinophagy [18–20]. In our study, we found that Pin1 promoted the K29/K48-linked ubiquitination of NCOA4. This dual-linkage ubiquitination pattern suggests a more complex regulatory mechanism than canonical K48-dependent degradation alone. K29-linked ubiquitination, an atypical linkage, participates in diverse cellular processes including protein interactions, vesicle biogenesis, viral regulation, and lysosomal degradation [55–57]. Although K29 linkage alone may not induce degradation, it often cooperates with K48 chains to regulate proteasomal turnover [58, 59]. Consistently, our findings demonstrate that Pin1-induced K29/K48-linked ubiquitination of NCOA4 promotes its proteasomal degradation.

In summary, this study reveals a critical mechanism through which Pin1 promotes ATC progression by inhibiting ferroptosis. Mechanistically, Pin1 binds to phosphorylated NCOA4, a key mediator of ferritinophagy, to induce K29/K48-linked ubiquitination and proteasomal degradation. This degradation event suppresses ferritinophagy-dependent iron release, thereby limiting ferroptotic sensitivity. Our findings establish Pin1 as a pivotal regulator of ferroptosis via NCOA4-mediated ferritinophagy and highlight Pin1 inhibition as a promising therapeutic strategy for ATC.

## Materials and methods

### Cell lines, transfection, and infection

KHM5M cells were cultured in RPMI-1640 medium supplemented with 10% fetal bovine serum (FBS). 8305 C cells were maintained in Minimum Essential Medium (MEM) with 10% FBS. HEK-293T cells were cultured in DMEM medium supplemented with 10% FBS. All cells were maintained at 37 °C in a humidified incubator with 5% CO₂. Cell transfection was carried out using Lipofectamine or polyethyleneimine (PEI, Polysciences) according to the manufacturer’s protocols. Lentiviral packaging (psPAX2/pMD2.G) and infection followed standard procedures. After infection, cells were selected with puromycin (1 μg/mL) or hygromycin (100 μg/mL) for ≥7 days. All cell lines were routinely tested for mycoplasma contamination and were confirmed to be contamination-free before experimentation. Unless otherwise stated, all in vitro experiments were independently repeated at least three times using biologically independent cultures.

### Antibodies and reagents

Anti-Pin1 antibody (A20718), anti-FTH1 antibody (A19544), anti-GPX4 antibody (A11243), anti-ATG7 antibody (A21895), anti-SQSTM1 antibody (A19700), anti-Ub antibody (A19686), anti-CDK2 antibody (A0294), anti-HA antibody (AE008), anti-GST antibody (AE001), and anti-GAPDH antibody (AC002) were obtained from Abclonal. Anti-NCOA4 antibody (10968-1-AP), anti-Pin1 antibody (68127-1-Ig), and anti-Flag antibody (66008-4-Ig) were obtained from Proteintech. Anti-phospho-(Ser) CDKs substrate antibody (2324) was obtained from Cell Signaling Technology. Anti-Ki67 (GB111499) was obtained from Servicebio. Anti-NCOA4-pS572 was generated by Abclonal with the peptides C-VLLN(pS)SPLQEE. Nec-1 (HY-15760), Z-VAD (HY-164388), Fer-1 (HY-100579), Deferoxamine (DFO, HY-B1625), MG132 (HY-13259), erastin (HY-15763), Ro-3306 (HY-12529), CVT-313 (HY-15339), ribociclib (HY-15777), abemaciclib (HY-16297A), and KPT-6566 (HY-123847) were obtained from MCE.

### DNA constructs and mutants

HA-CDK1, HA-CDK2, HA-CDK4, HA-CDK6, HA-Pin1, Flag-NCOA4 were cloned into mammalian expression pCDNA3-HA vectors. pCMV-GST-Pin1 was cloned into mammalian expression pCMV-GST vectors. The GST-NCOA4 bacterial expression construct was generated by cloning human NCOA4 into the pGEX-4T1 vector. pLenti-hygro-NCOA4 was cloned into mammalian expression pLenti-hygro vectors. NCOA4-S572A, NCOA4-S572D, and Pin1 mutant (W34A/K63A) were obtained via Q5 Site-Directed Mutagenesis Kit (New England Biolabs) according to the manufacturer’s instructions and the sequences were verified by DNA sequencing. Specific shRNA primers were cloned into pLKO.1-puro lentiviral vectors, human Pin1 target sequence #1 5′-CCATTTGAAGACGCCTCGTT-3′, human Pin1 target sequence #2 5′-AGAAGCCATTTGAAGACGCCT-3′, human Pin1 target sequence #3 5′-CCAGAAGATCAAGTCGGGAGA-3′, human CDK2 target sequence #1 5′-GAGCTTAACCATCCTAATATT-3′, human CDK2 target sequence #2 5′-ACGGAGCTTGTTATCGCAAAT-3′, human CDK2 target sequence #3 5′-CGTACGGAGTTGTGTACAAAG-3′.

### Thyroid cancer tissues and immunohistochemical staining

All thyroid cancer tissues and matched adjacent normal tissues were obtained from The First Affiliated Hospital of Sun Yat-sen University and the Cancer Hospital of Shantou University Medical College. The use of human specimens was approved by the Institutional Ethics Committee of the First Affiliated Hospital of Sun Yat-sen University (Approval No. 2019-275) and the Ethics Committee of the Cancer Hospital of Shantou University Medical College (Approval No. 2022093), and informed consent was obtained from all patients. Immunohistochemical staining and scoring were independently assessed by two blinded investigators to minimize observer bias. Tumor xenografts and surgical specimens were fixed in 10% neutral-buffered formalin, embedded in paraffin, and sectioned at 4 μm thickness. For IHC staining, tissue sections were incubated with 3% hydrogen peroxide for 15 min at room temperature to quench endogenous peroxidase activity, followed by blocking with 5% normal goat serum for 1 h at room temperature. Sections were then incubated overnight at 4 °C with primary antibodies against FTH1, Pin1, NCOA4, phosphorylated NCOA4 (pNCOA4), and Ki67. The next day, sections were incubated with monoclonal mouse anti-rabbit immunoglobulin (Dako #M0737) for 1 h at room temperature, followed by incubation with the Envision+ System-HRP Labeled Polymer Anti-Rabbit (Dako #K4003) for 30 min. Specific immunoreactivity was visualized using a DAB chromogen kit (Dako #K3468) and counterstained lightly with hematoxylin.

### Immunoprecipitation (IP), GST pull-down (PD) assay and western blot

Cells were lysed in EBC buffer (50 mM Tris, pH 7.5; 120 mM NaCl; 0.5% NP-40) supplemented with protease inhibitors (PhosSTOP, Roche) and phosphatase inhibitors (Calbiochem Phosphatase Inhibitor Cocktail Sets I and II). Total protein concentrations were determined using the bicinchoninic acid (BCA) protein assay (Pierce™ BCA Protein Assay Kit, #23225). Equal amounts of protein lysates were resolved by 10% SDS-PAGE and transferred to Immobilon-P PVDF membranes (Millipore, MA, USA). The membranes were incubated with primary antibodies overnight at 4 °C, followed by incubation with appropriate secondary antibodies at room temperature for 1 h. Protein bands were detected using an enhanced chemiluminescence (ECL) detection system as described previously. For immunoprecipitation assays, whole-cell lysates were incubated with the indicated primary antibodies (1–2 μg) for 3–4 h at 4 °C, followed by incubation with Protein A/G Sepharose beads (GE Healthcare) overnight at 4 °C. For GST pull-down assays, lysates were incubated with glutathione-Sepharose 4B beads (GE Healthcare) for 2 h at 4 °C. Immunocomplexes were washed four times with NETN buffer (20 mM Tris, pH 8.0; 150 mM NaCl; 1 mM EDTA; 0.5% NP-40) and analyzed by SDS-PAGE followed by immunoblotting with the indicated antibodies.

### Immunostaining (IF)

Cells cultured on confocal microscopy dishes were fixed with 4% paraformaldehyde, followed by blocking with 10% normal goat serum. The cells were then incubated with primary antibodies against NCOA4 and Pin1, washed three times with PBS, and subsequently incubated with Alexa Fluor 488- (green) and Alexa Fluor 647- (red) conjugated secondary antibodies. Nuclear DNA was counterstained with DAPI. Images were acquired using a confocal laser scanning microscope.

### Purification of GST-tagged proteins from bacteria

Recombinant GST-tagged NCOA4 protein was produced by transforming E. coli BL21 (DE3) cells with the pGEX-4T1-NCOA4 plasmid. Overnight starter cultures grown at 37 °C were diluted 1:100 into fresh medium and further incubated at 37 °C until reaching an optical density (OD600) of 0.8. Protein expression was then induced with 0.1 mM isopropyl β-D-1-thiogalactopyranoside (IPTG) at 16 °C for 16 h with vigorous shaking. Bacterial pellets were harvested and resuspended in EBC buffer, followed by sonication to lyse the cells. The lysates were centrifuged to remove insoluble material and cell debris. The supernatant was incubated with 50 μL of 50% Glutathione-Sepharose 4B slurry (Pierce) for 3 h at 4 °C with gentle rotation. Beads were then washed three times with PBS to remove non-specific proteins. Purified GST-NCOA4 protein was either stored at 4 °C in PBS containing 10% glycerol or eluted using elution buffer as required. Protein purity was assessed by Coomassie Brilliant Blue staining following SDS-PAGE, and concentration was determined using BSA as a standard.

### In vivo ubiquitination assay

In vivo ubiquitination assays were conducted as previously described [24]. Briefly, HEK293T cells were co-transfected with His-tagged ubiquitin (His-Ub) and the indicated expression constructs. Twenty-four h post-transfection, cells were treated with 10 μM MG132 for 12 h to inhibit proteasomal degradation. Cells were then washed twice with cold PBS and lysed in denaturing buffer A (6 M guanidine-HCl, 0.1 M Na₂HPO₄/NaH₂PO₄, and 10 mmol/L imidazole, pH 8.0), followed by sonication. Lysates were clarified by high-speed centrifugation, and the resulting supernatants were incubated with nickel-nitrilotriacetic acid (Ni-NTA) agarose beads (QIAGEN) for 3 h at room temperature with rotation. The beads were subsequently washed twice with buffer A, twice with buffer A/TI (1 part buffer A mixed with 3 parts buffer TI), and twice with buffer TI (25 mmol/L Tris-HCl and 20 mmol/L imidazole, pH 6.8) to remove non-specific binding proteins. The bound ubiquitinated proteins were eluted by boiling in SDS loading buffer and analyzed by SDS-PAGE (8% gel) followed by immunoblotting using the appropriate antibodies.

### In vitro kinase assay

The in vitro kinase assay was performed as previously described [24]. In brief, 1 μg of bacterially purified GST-tagged NCOA4 fusion protein was incubated with immunoprecipitated CDK2, obtained from HEK-293T cells transfected with various mutant forms of CDK2, in kinase reaction buffer (50 mM Tris-HCl, pH 7.5, 10 mM MgCl₂, 1 mM DTT, 100 μM ATP). The reaction was carried out at 30 °C for 30 min and terminated by the addition of 0.1 mmol/L EDTA. Reaction products were separated by SDS-PAGE and analyzed by immunoblotting using an anti-NCOA4 antibody.

### Peptide synthesis and dot immunoblot assay

Synthetic peptides corresponding to NCOA4-S572 wild-type (WT) and phosphorylated (Phospho) sequences were obtained from Abclonal. The sequences were as follows:

NCOA4-S572-WT: C- VLLNSPLQEE

NCOA4-S572-Phospho: C- VLLN(pS)SPLQEE

Peptides were diluted to a concentration of 2 mg/mL and spotted onto nitrocellulose membranes at the following doses: 0.01, 0.03, 0.10, and 0.30 μg. After air drying, the membranes were blocked with 5% non-fat milk in TBST buffer and subjected to immunoblot analysis.

### Mass spectrometry

Flag-IP products from HEK293T cells transfected with Flag-NCOA4 ± HA-CDK2 were resolved by SDS-PAGE and stained with Coomassie. Bands were reduced (10 mM DTT), alkylated (55 mM iodoacetamide), and digested with trypsin. Peptides were analyzed by LC-MS/MS on a Q Exactive HF Orbitrap with HCD fragmentation coupled to an EASY-nLC II. Spectra were searched against the UniProt Homo sapiens reference proteome (UP000005640) using Mascot 2.5.1 and Scaffold; FDR was controlled at 1% (PSM and protein).

### Quantitative proteomics

Proteins from Pin1-knockdown and control KHM5M cells were processed by reduction (5 mM DTT, 56 °C, 30 min), alkylation (11 mM iodoacetamide, 15 min, dark), dilution to <2 M urea, and trypsin digestion (1:50 overnight + 1:100 for 4 h). Peptides were separated on a nanoElute UHPLC (2–22% B over 76 min; 22–35% over 6 min; to 90% in 4 min; hold 4 min; 450 nL/min) and analyzed on a timsTOF Pro in PASEF mode (Top10; dynamic exclusion 24 s). Raw data were processed in MaxQuant (v1.6.15.0), searched against the UniProt Homo sapiens proteome (UP000005640) with a reverse decoy; Trypsin/P, up to two missed cleavages; precursor tolerance 20 ppm (first) and 4.5–10 ppm (main); fragment tolerance 0.05–0.1 Da; fixed Carbamidomethyl-C; variable Acetyl-N-term and Oxidation-M. FDR was 1% at PSM and protein levels.

### Ferrous Iron detection

Intracellular ferrous iron (Fe²⁺) levels were quantified using a colorimetric Iron Assay Kit (Elabscience, Wuhan, China) following the manufacturer’s instructions. Briefly, cells were harvested, lysed in buffer solution on ice for 10 min, and centrifuged at 15,000 × *g* for 10 min at 4 °C to remove insoluble debris. The supernatant was collected, and 100 μL of chromogenic solution was added to each sample, followed by incubation at 37 °C for 10 min. Absorbance was measured at 593 nm using a microplate reader (Thermo Fisher Scientific).

### Lipid peroxidation detection

To assess lipid peroxidation and cell death, 1 × 10⁶ cells suspended in 2 mL of complete medium were seeded in 6-well plates and incubated overnight. Cells were then treated with the indicated concentrations of KPT-6566 for 24 h. For lipid peroxidation analysis, cells were incubated with 2 μM C11-BODIPY 581/591 (Invitrogen, Carlsbad, CA, USA) for 30 min at 37 °C. After staining, cells were washed twice with PBS, harvested, and analyzed by flow cytometry. Data were processed using FlowJo software.

### MDA detection

Intracellular malondialdehyde (MDA) levels were determined using an MDA Detection Kit (Solarbio Life Science, Beijing, China) according to the manufacturer’s instructions. Briefly, cells were lysed on ice in MDA Lysis Buffer and centrifuged at 12,000 × *g* for 15 min to remove insoluble debris. The resulting supernatants were incubated with thiobarbituric acid (TBA) solution at 100 °C for 1 h, cooled to room temperature, and centrifuged again at 10,000 × *g* for 10 min. Absorbance was measured at 532 nm using a microplate reader (Thermo Fisher Scientific) to quantify MDA levels.

### LDH releasing assay

LDH release was measured using a commercial LDH Assay Kit (C0017, Beyotime, Shanghai, China) according to the manufacturer’s instructions. Briefly, culture medium was transferred to a new 96-well plate and mixed with the Reaction Mixture. After incubation at room temperature for 30 min, the reaction was terminated by adding the Stop Solution. Absorbance was measured at 532 nm using a microplate reader (Thermo Fisher Scientific) to quantify LDH release.

### Hydrogen peroxide (H_2_O_2_) detection

Intracellular hydrogen peroxide (H₂O₂) levels were measured using the CheKine™ Micro Hydrogen Peroxide (H₂O₂) Assay Kit (KTB1041, Abbkine, China) according to the manufacturer’s instructions. Briefly, cells were harvested after the indicated treatments and lysed in the assay buffer. After centrifugation to remove insoluble debris, the supernatants were collected and mixed with the reaction buffer and H₂O₂ detection reagents in a 96-well plate. Following incubation at room temperature, the absorbance was measured at 560 nm using a microplate reader (Thermo Fisher Scientific). H₂O₂ concentrations were calculated according to the standard curve and expressed as relative values normalized to the control group.

### JC-1 staining

Treated KHM5M cells were seeded into confocal dishes and stained using the JC-1 Mitochondrial Membrane Potential Detection Kit (Beyotime Biotechnology), according to the manufacturer’s instructions. Briefly, cells were incubated with JC-1 staining solution at 37 °C for 30 min. After incubation, cells were washed twice with PBS and imaged using a Leica SP8 confocal microscope. In cells with intact mitochondrial membrane potential (Δψm), JC-1 accumulates in the mitochondrial matrix to form aggregates, which emit red fluorescence (emission at 590 nm). In contrast, depolarized mitochondria maintain JC-1 in its monomeric form, emitting green fluorescence (emission at 529 nm). Mitochondrial membrane potential was assessed by calculating the JC-1 red (aggregate) to green (monomer) fluorescence ratio, with a lower red/green ratio indicating mitochondrial depolarization.

### FerroOrange staining

To visualize intracellular labile ferrous iron (Fe²⁺), cells were stained with FerroOrange (Dojindo Laboratories) following the manufacturer’s protocol. Briefly, cells cultured on confocal dishes were subjected to the indicated treatments, washed twice with warm PBS, and incubated with 1 μM FerroOrange working solution at 37 °C for 30 min in the dark. After incubation, cells were washed with PBS and counterstained with Hoechst 33342 to visualize nuclei. Fluorescence images were captured using a Leica SP8 confocal microscope. All images were acquired using identical laser power, detector gain, and exposure settings. The fluorescence intensity of FerroOrange was analyzed as an indicator of intracellular Fe²⁺ accumulation.

### Cell viability assay (CCK-8)

Cell viability was assessed using the Cell Counting Kit-8 (CCK-8; C0038, Beyotime, Shanghai, China) following the manufacturer’s protocol. Briefly, KHM5M and 8305 C cells were seeded into 96-well plates at a density of 1000 cells per well and treated with the indicated concentrations of erastin for 24 h. After treatment, CCK-8 solution was added to the culture medium at a final concentration of 10%, followed by incubation at 37 °C for 2 h. The optical density (OD) was measured at 450 nm using a microplate reader (Thermo Fisher Scientific). Cell viability was expressed as the percentage of OD relative to the control group.

### Colony formation

Cells were seeded into 6-well plates at a density of 300 or 600 cells per well and cultured for 12–20 days until visible colonies formed. Colonies were washed with PBS, fixed with 4% paraformaldehyde for 20 min, and stained with 0.4% crystal violet in 20% ethanol for 20 min. Plates were then rinsed with water, air-dried, and the number of colonies was manually counted and quantified.

### Xenograft models and in vivo treatments

Mouse xenograft assays were conducted as previously described. All animal experiments were approved by the Institutional Animal Care and Use Committee of Sun Yat-sen University (Approval No. SYSU-IACUC-2021-000647). Female BALB/c nude mice (4 weeks old, *n* = 8 mice per group) were obtained from Guangdong GemPharmatech. A total of 3 × 10⁶ KHM5M cells stably expressing the target gene were resuspended in Matrigel and injected subcutaneously into the flank region. When tumors reached a volume of 50–100 mm³, mice were treated with vehicle control and KPT-6566 (20 mg/kg), administered three times per week. Randomization was performed to minimize allocation bias. Investigators measuring tumor size were blinded to treatment groups whenever feasible. No animals were excluded after randomization unless pre-established humane endpoints or unrelated health complications occurred. Tumor volume measurements represent biological replicates derived from individual mice. Tumor growth was monitored every three days using calipers, and tumor volume was calculated using the formula: Volume = (length × width²) × 0.5, where length is the longest and width is the shortest tumor diameter. Mice were euthanized when tumor volume exceeded 2000 mm³.

### Quantification and statistical analyses

All experiments were independently repeated at least three times unless otherwise specified. Sample sizes were selected based on prior experience with comparable mechanistic cancer biology studies and preliminary experimental reproducibility. In vitro assays were independently repeated at least three times using biologically independent samples. For animal studies, eight mice per group were used, which is consistent with standard xenograft experimental designs sufficient to detect biologically meaningful differences in tumor growth. Exact sample sizes (n) are indicated in the corresponding Fig. legends. Quantitative data are presented as mean ± standard deviation (SD), unless otherwise indicated. Statistical analyses were performed using GraphPad Prism version 10.0. Comparisons between two groups were performed using unpaired two-tailed Student’s *t* tests. Comparisons among multiple groups were performed using one-way ANOVA followed by Tukey’s multiple-comparison test. For experiments involving multiple variables or time-course analyses, two-way ANOVA followed by Sidak’s post hoc correction was applied. For experiments with small sample sizes, individual data points were plotted whenever possible. Statistical assumptions for parametric testing, including approximate normal distribution and comparable variance, were assessed visually. Exact *P* values, sample size (n), and statistical tests used are reported in the corresponding Fig. legends. *P* < 0.05 was considered statistically significant. No statistical method was used to predetermine sample size; however, sample sizes were selected based on prior studies and laboratory experience to ensure reproducibility and sufficient statistical power. No data were excluded from analysis unless a technical failure occurred.

### Reporting summary

All representative images shown in the Figures were derived from at least three independent experiments yielding similar results. Raw data supporting the findings of this study are available from the corresponding author upon reasonable request. Full uncropped immunoblot images are provided in the Supplementary Information.

## Supplementary information

Supplementary Fig.

Full and uncropped western blots

## Data Availability

All data generated or analyzed during this study are included in this published article and its supplementary information files. Full uncropped immunoblot and gel images are provided in the Supplementary Information. The mass spectrometry proteomics data have been deposited to the ProteomeXchange Consortium (https://proteomecentral.proteomexchange.org) via the iProX partner repository with the dataset identifier PXD077217 [60, 61].
