# Supplementary material for: Pin1 targets phosphorylated NCOA4 for K29/K48-linked ubiquitination to suppress ferritinophagy and ferroptosis in anaplastic thyroid cancer
Source: Cell Death Dis. 2026 Jul 1;17(1):815. doi: 10.1038/s41419-026-09058-5 (PMC13586214; doi:10.1038/s41419-026-09058-5)

2C

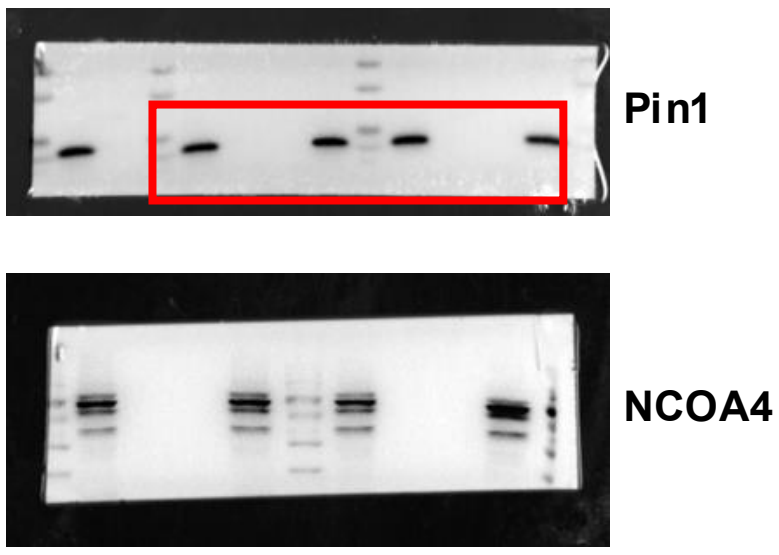

2D

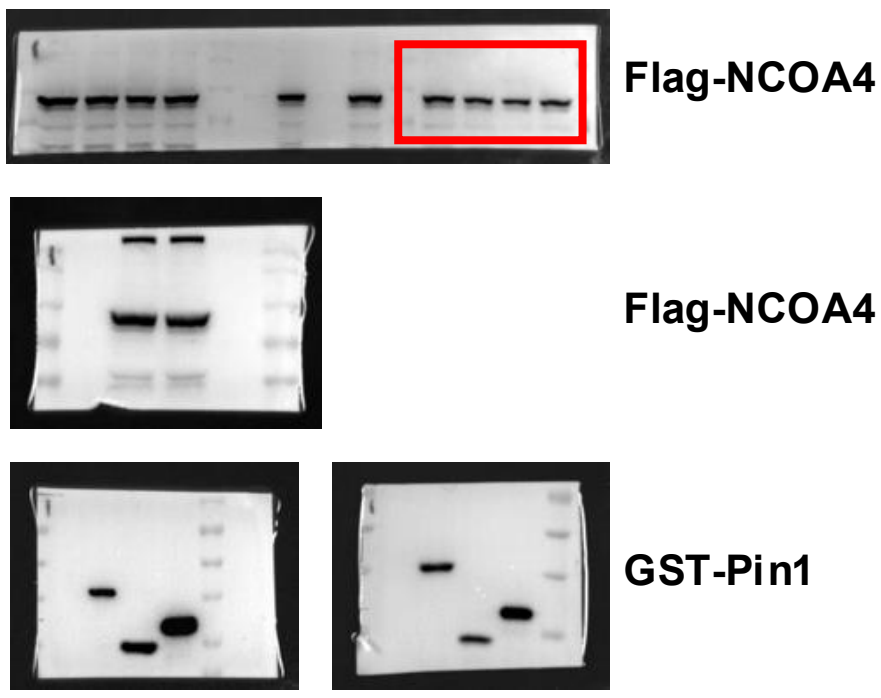

2E

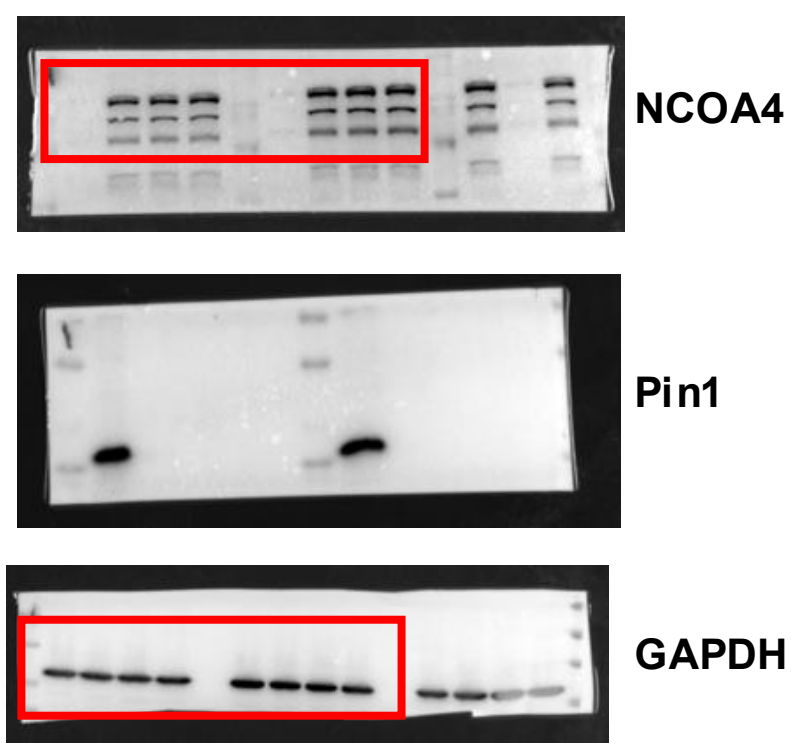

2G

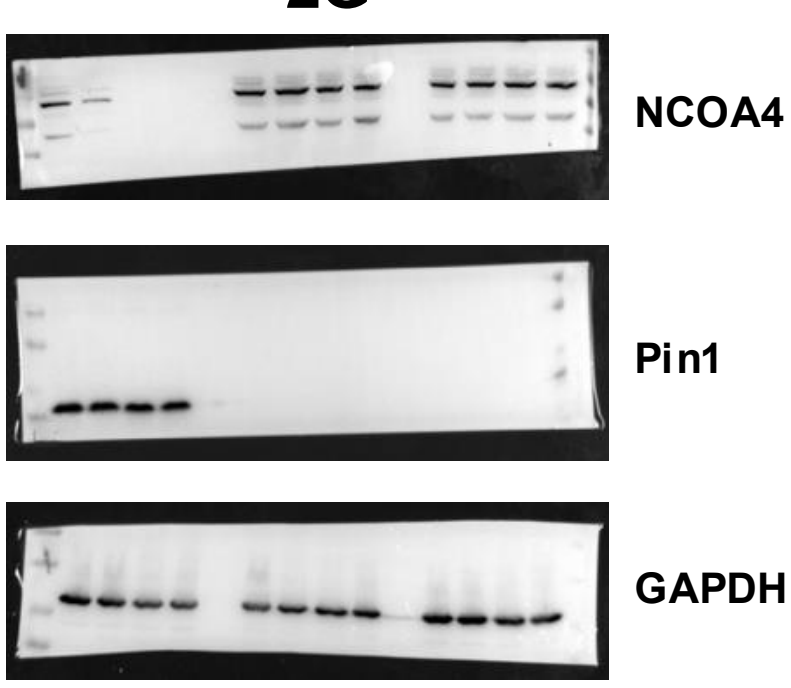

2H

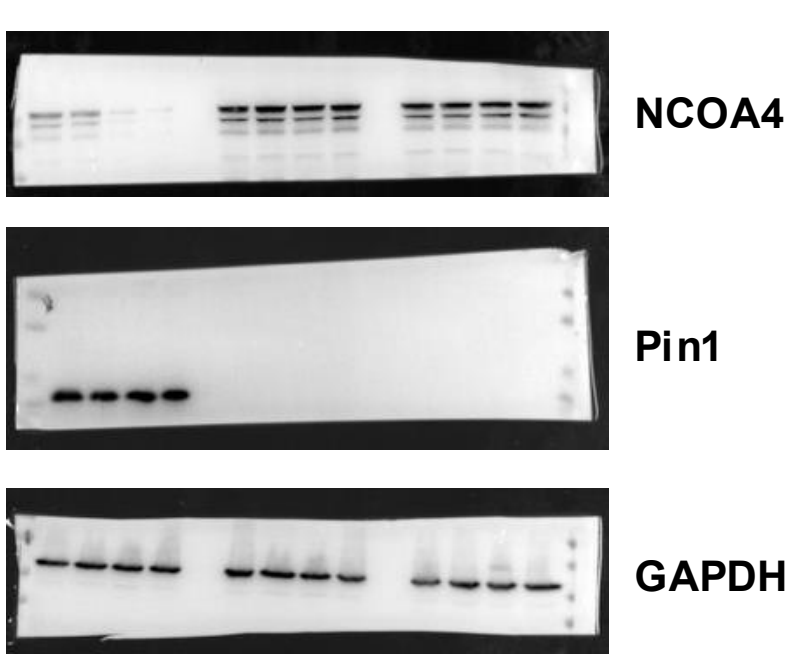

2K

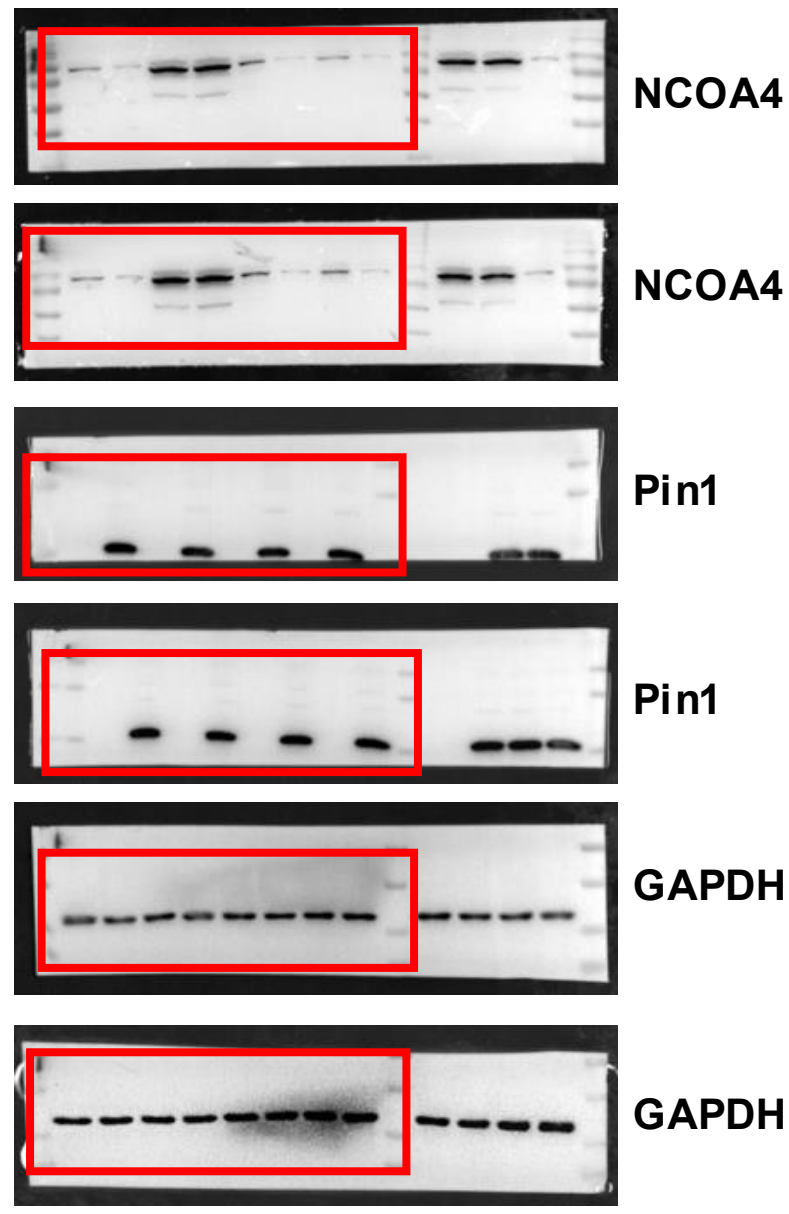

3A

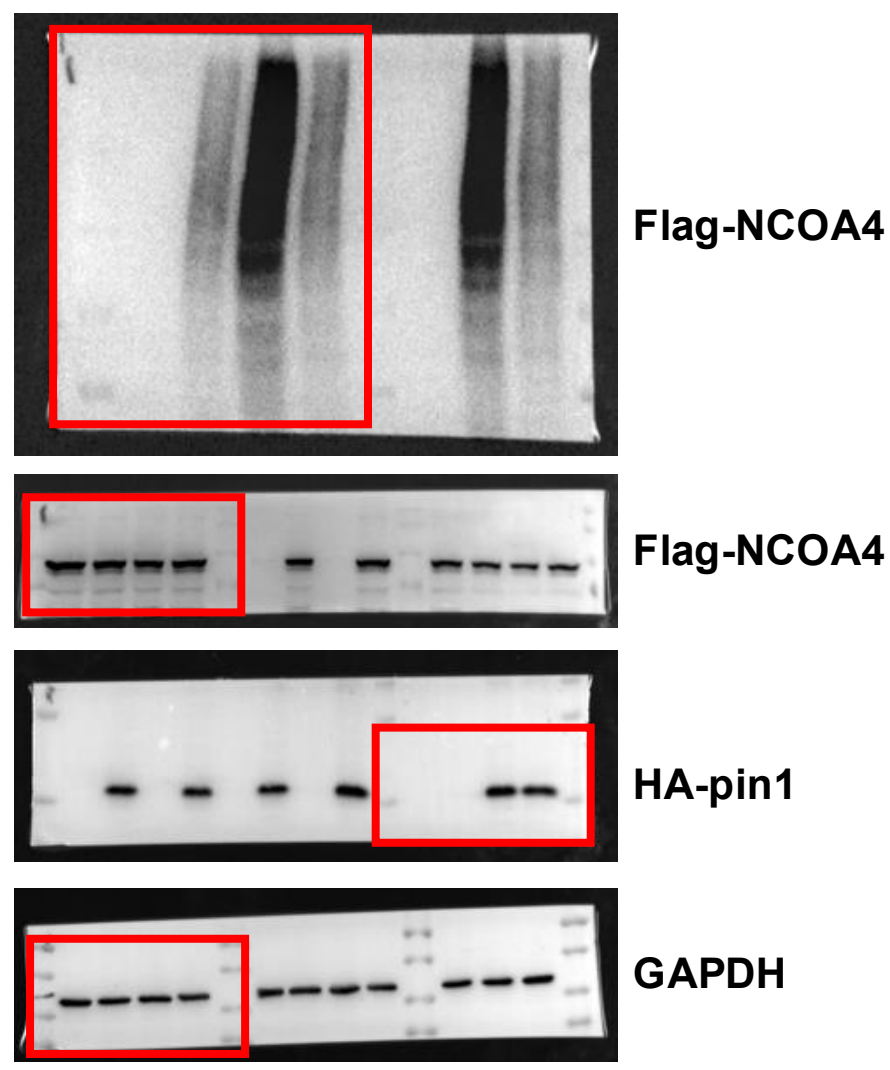

3B

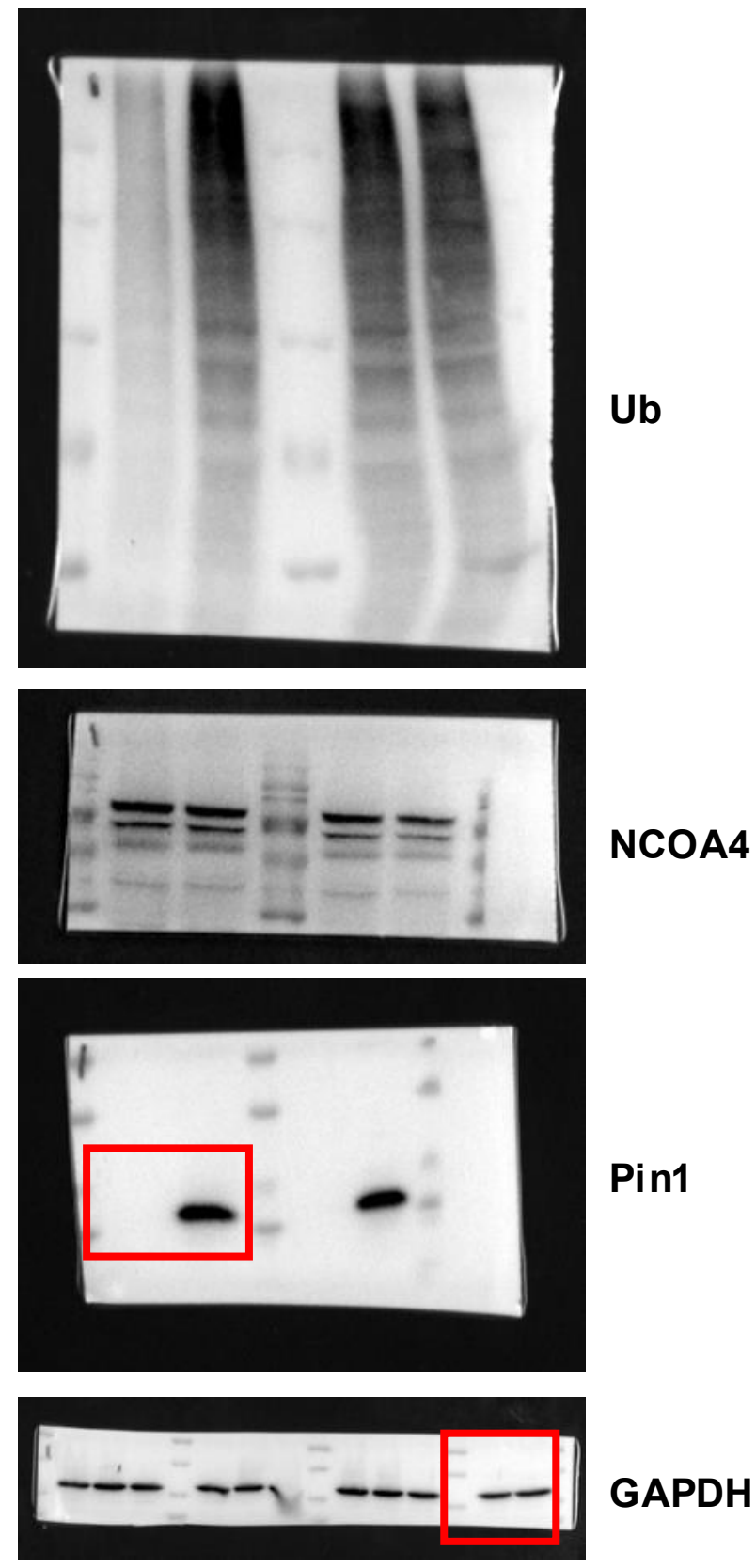

3C

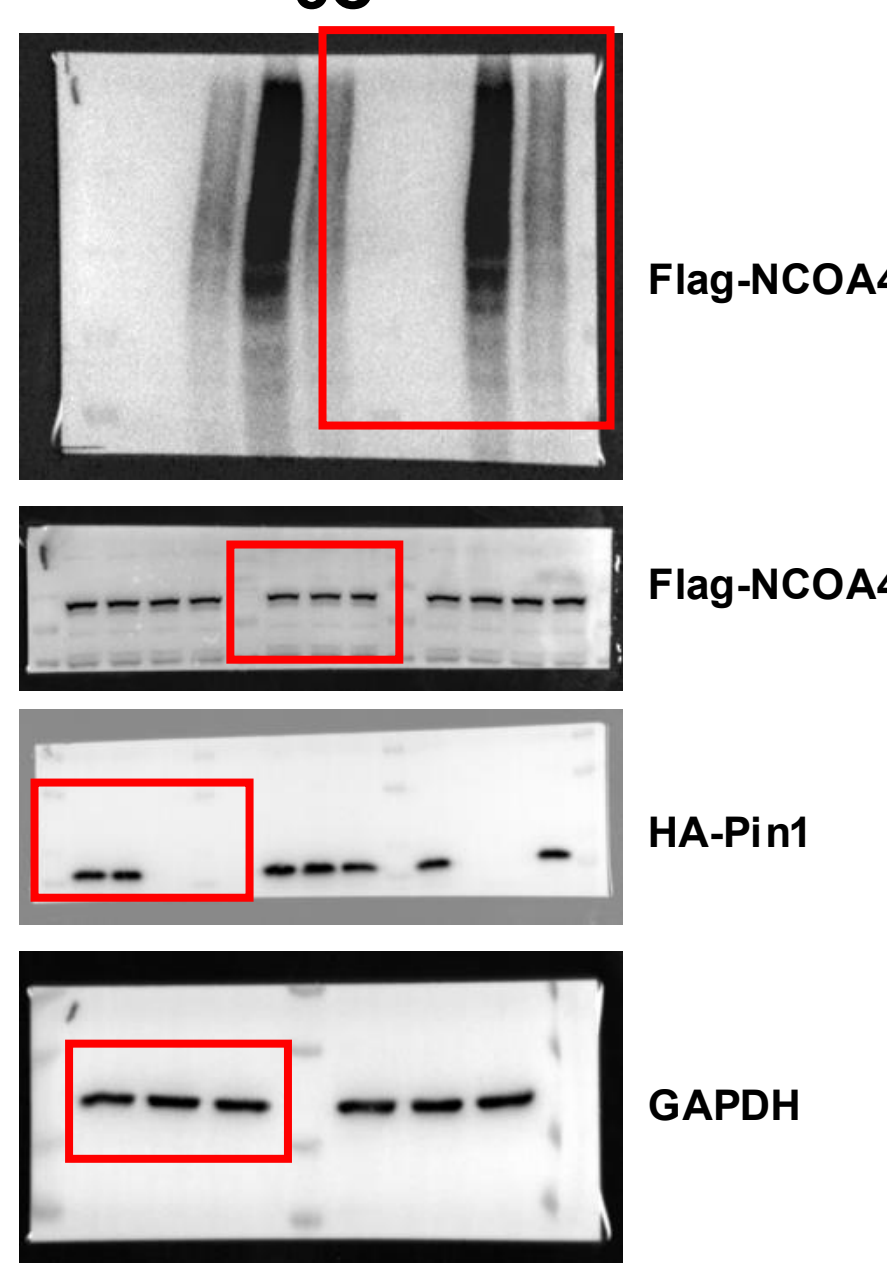

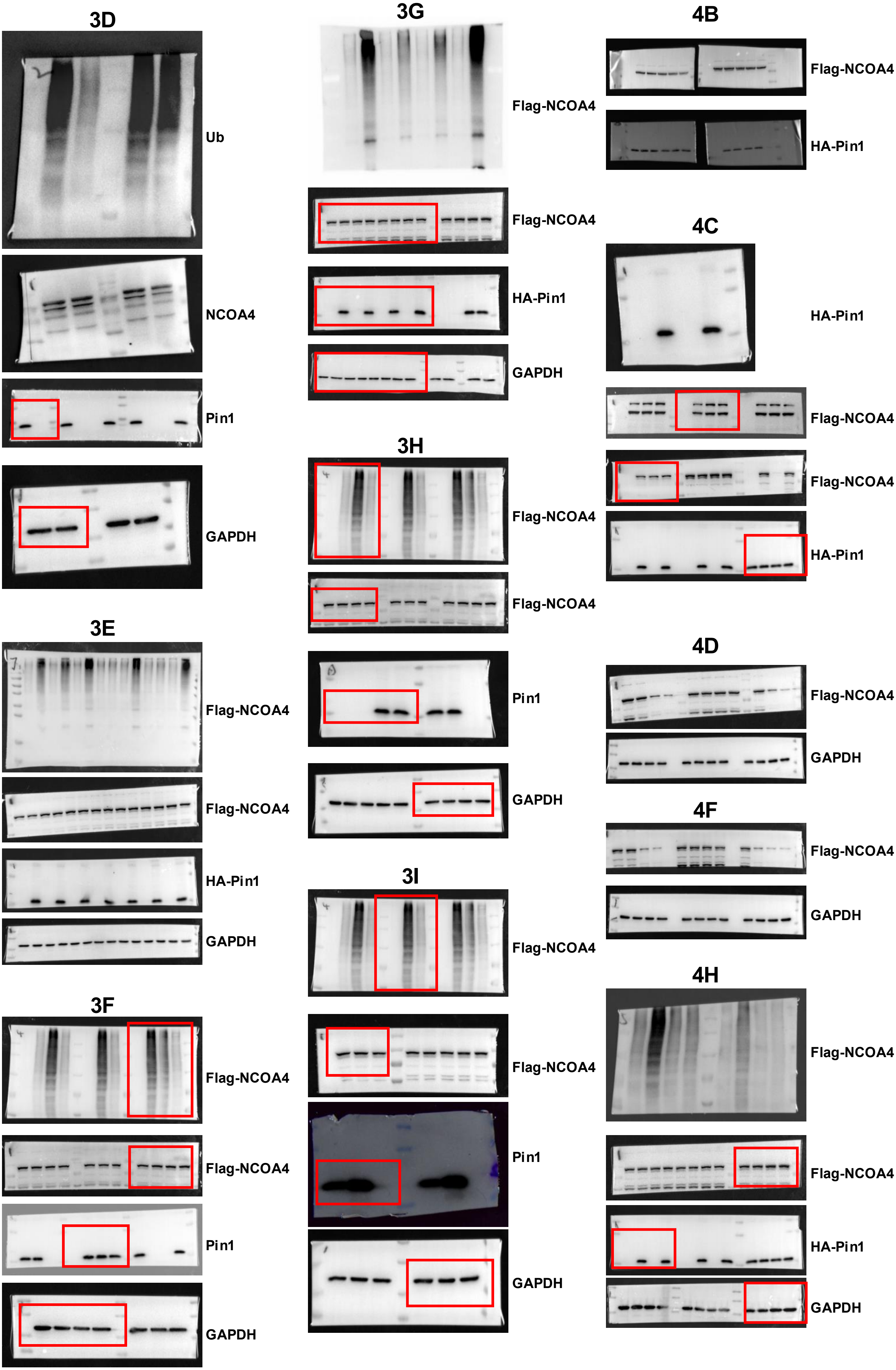
3D
Ub
NCOA4
Pin1
GAPDH
3E
Flag-NCOA4
Flag-NCOA4
HA-Pin1
GAPDH
3F
Flag-NCOA4
Flag-NCOA4
Pin1
GAPDH
3G
Flag-NCOA4
Flag-NCOA4
HA-Pin1
GAPDH
3H
Flag-NCOA4
Flag-NCOA4
Pin1
GAPDH
3I
Flag-NCOA4
Flag-NCOA4
Pin1
GAPDH
4B
Flag-NCOA4
HA-Pin1
4C
HA-Pin1
Flag-NCOA4
Flag-NCOA4
HA-Pin1
4D
Flag-NCOA4
GAPDH
4F
Flag-NCOA4
GAPDH
4H
Flag-NCOA4
Flag-NCOA4
HA-Pin1
GAPDH

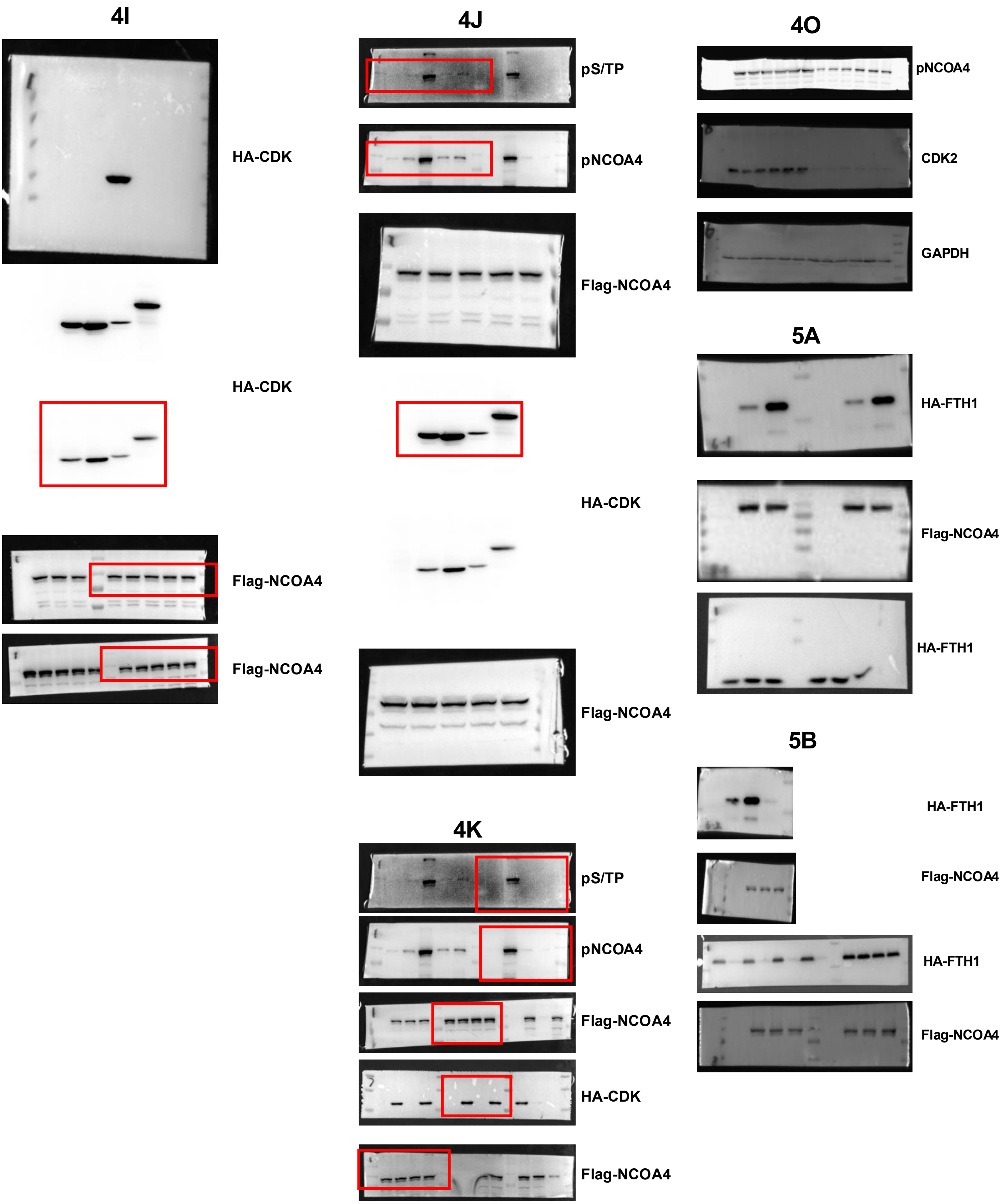
4I
HA-CDK
HA-CDK
Flag-NCOA4
Flag-NCOA4
4J
pS/TP
pNCOA4
Flag-NCOA4
HA-CDK
Flag-NCOA4
4K
pS/TP
pNCOA4
Flag-NCOA4
HA-CDK
Flag-NCOA4
4O
pNCOA4
CDK2
GAPDH
5A
HA-FTH1
Flag-NCOA4
HA-FTH1
5B
HA-FTH1
Flag-NCOA4
HA-FTH1
Flag-NCOA4

5P

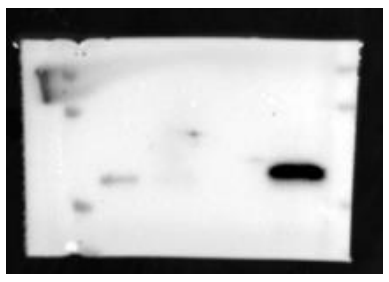
FTH1

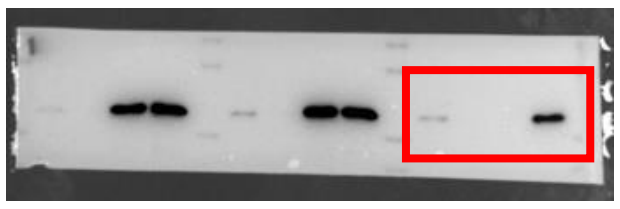
FTH1

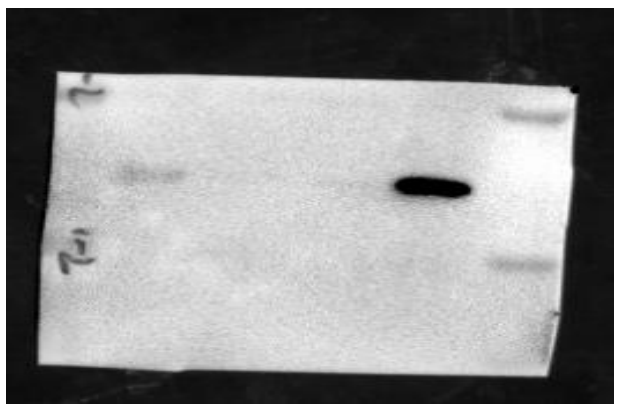
GPX4

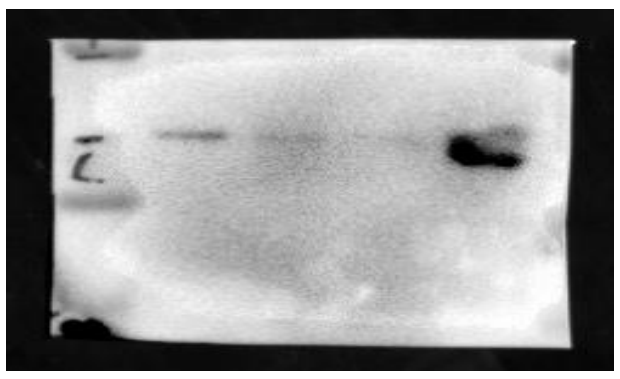
GPX4

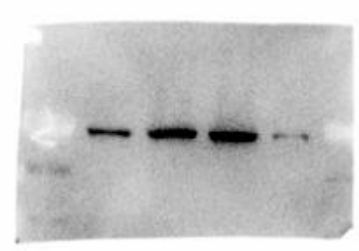
ATG7

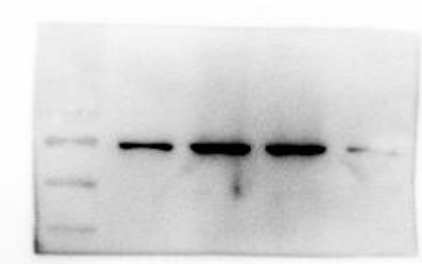
ATG7

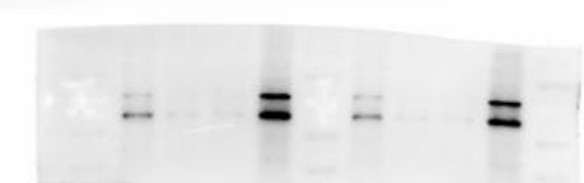
SQSTM1

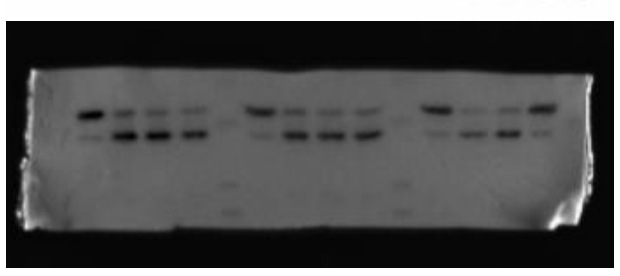
LC3

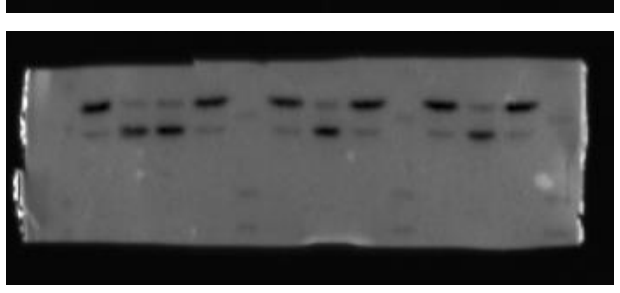
LC3

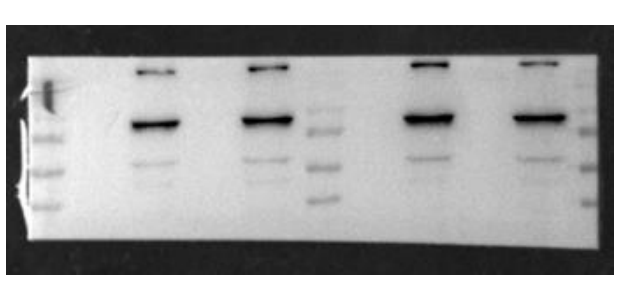
pNCOA4

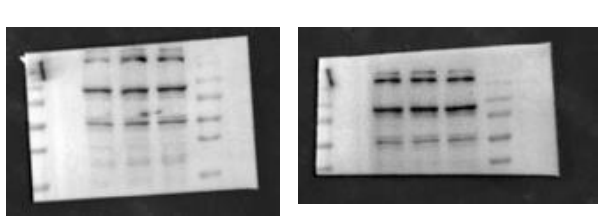
Flag-NCOA4

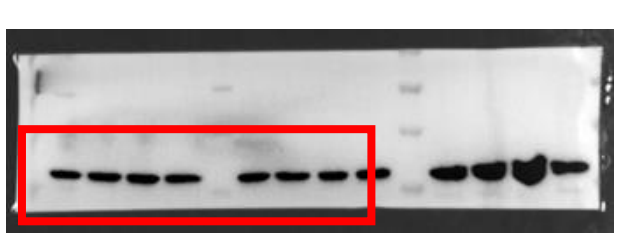
GAPDH

6K

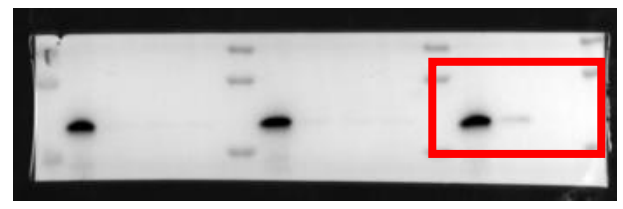
FTH1

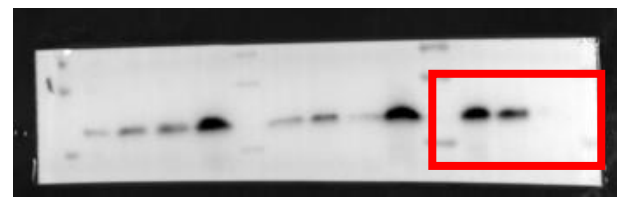
FTH1

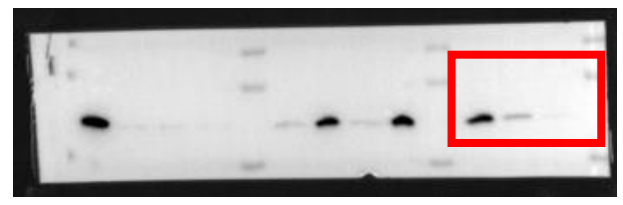
GPX4

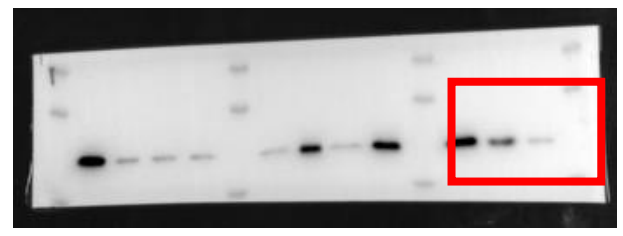
GPX4

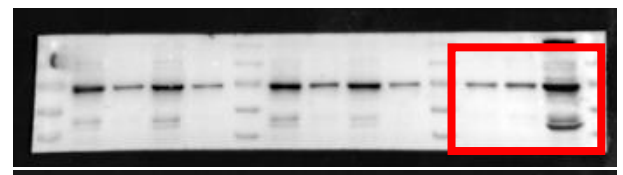
ATG7

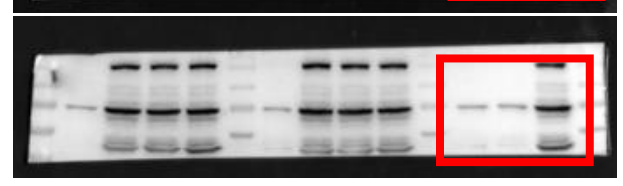
ATG7

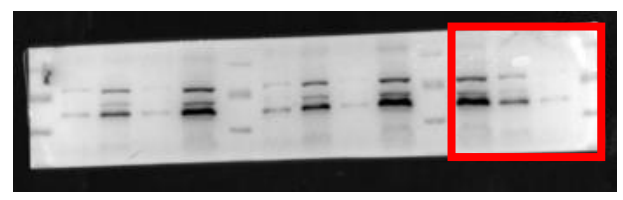
SQSTM1

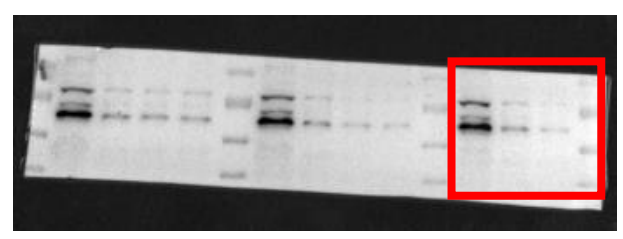
SQSTM1

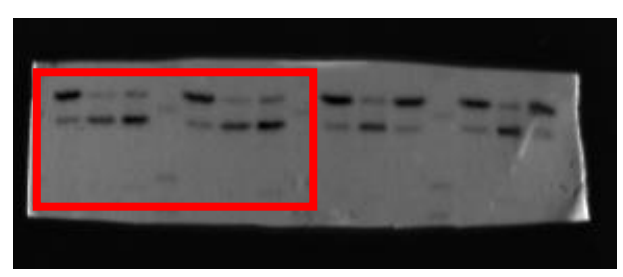
LC3

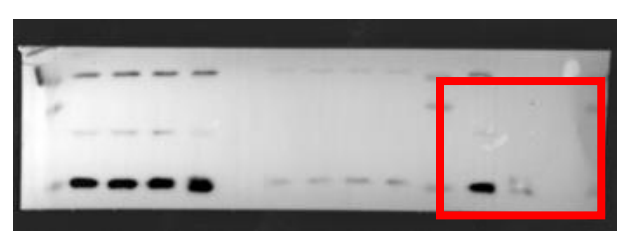
Pin1

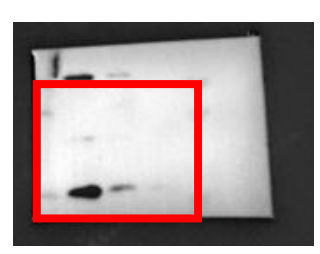
Pin1

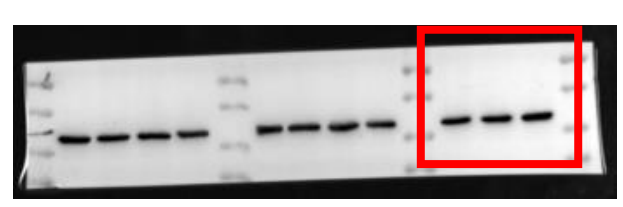
GAPDH

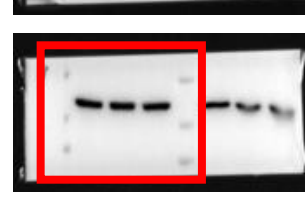
GAPDH

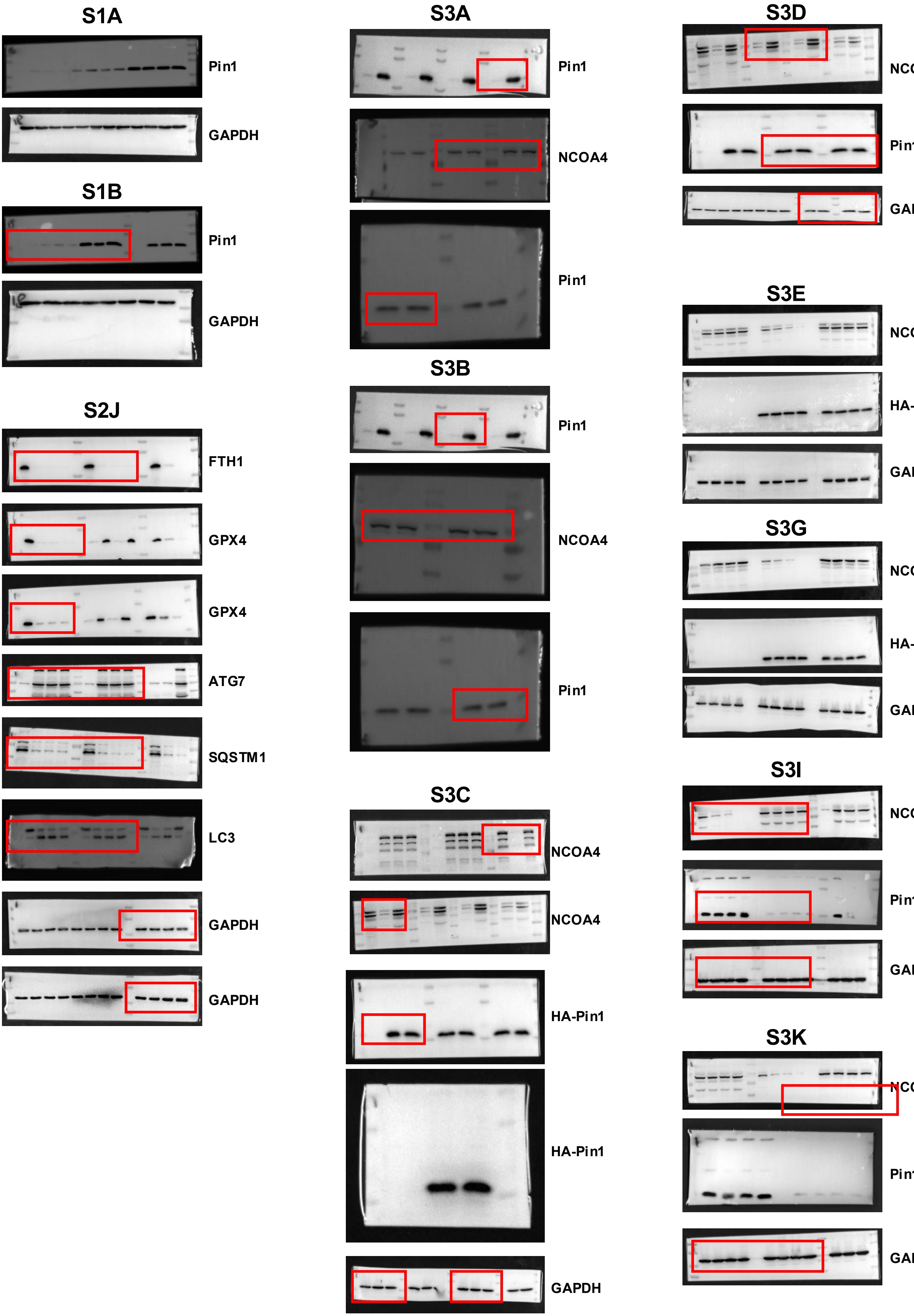
S1A
Pin1
GAPDH
S1B
Pin1
GAPDH
S2J
FTH1
GPX4
GPX4
ATG7
SQSTM1
LC3
GAPDH
GAPDH
S3A
Pin1
NCOA4
Pin1
S3B
Pin1
NCOA4
Pin1
S3C
NCOA4
NCOA4
HA-Pin1
HA-Pin1
GAPDH
S3D
S3E
S3G
S3I
S3K

S4M

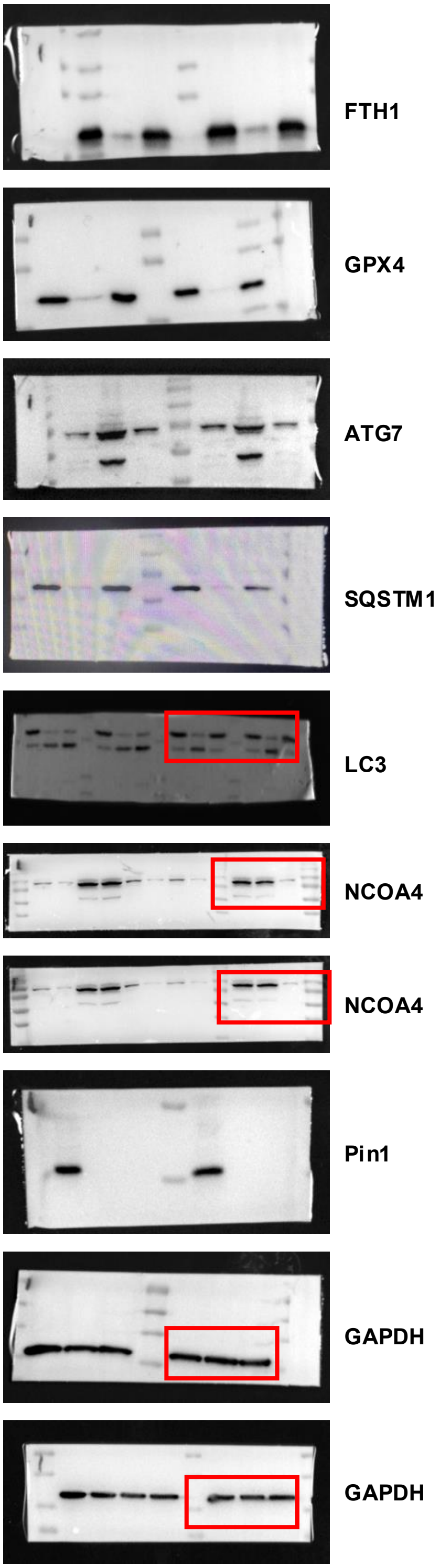

S6A

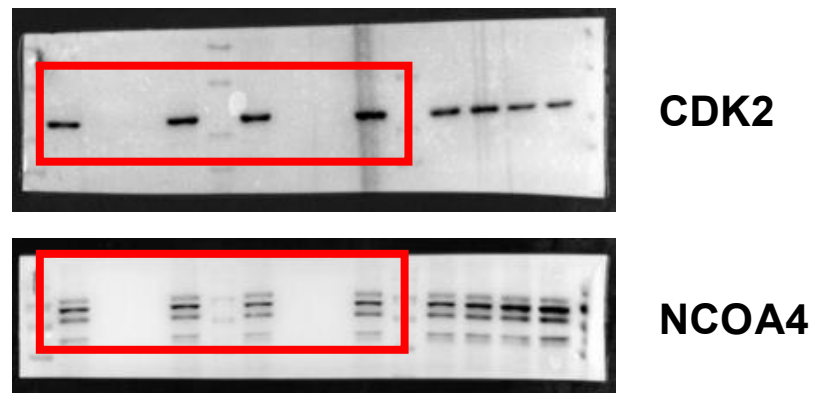

S6D

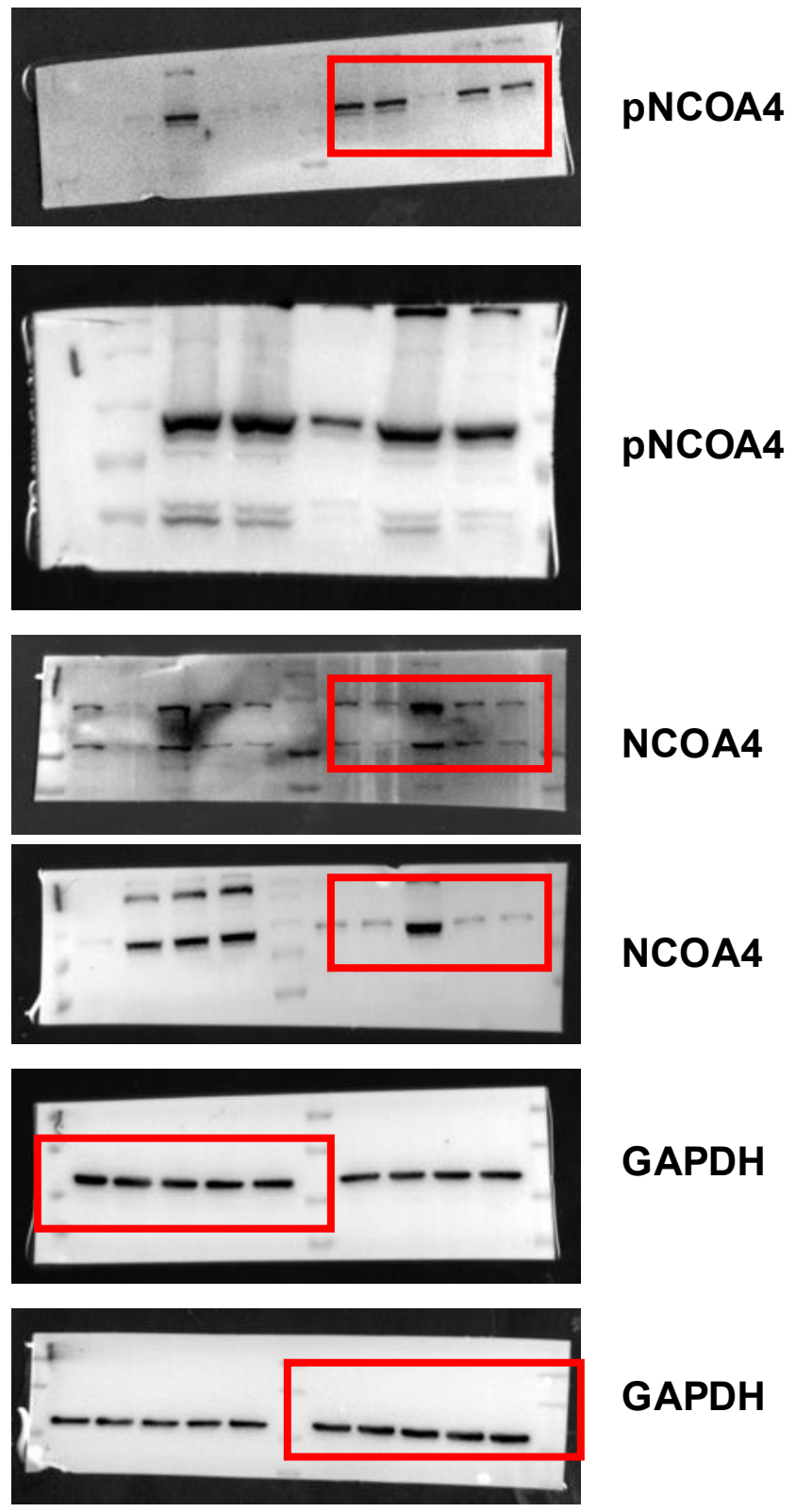

S6E

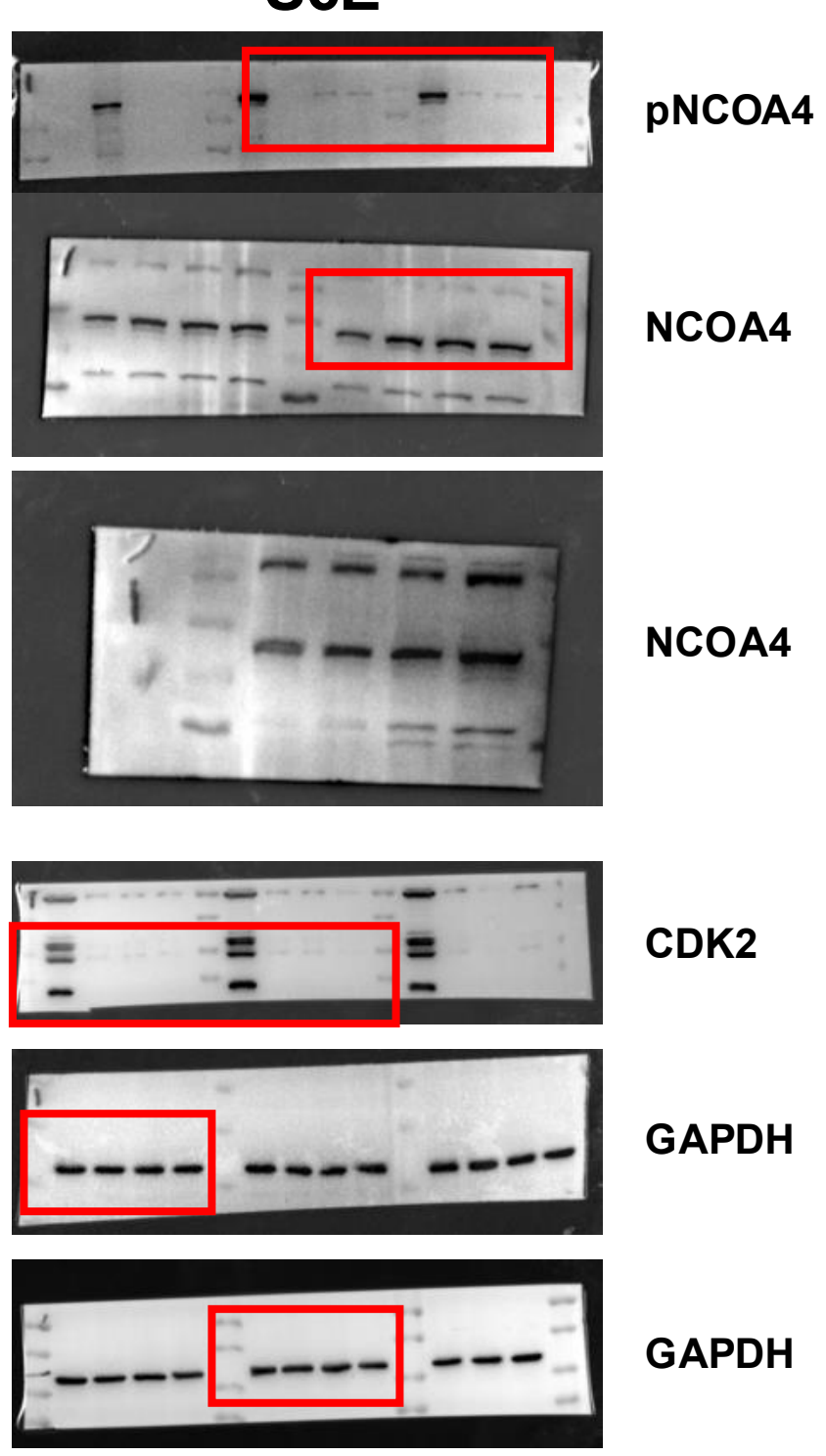

S6F

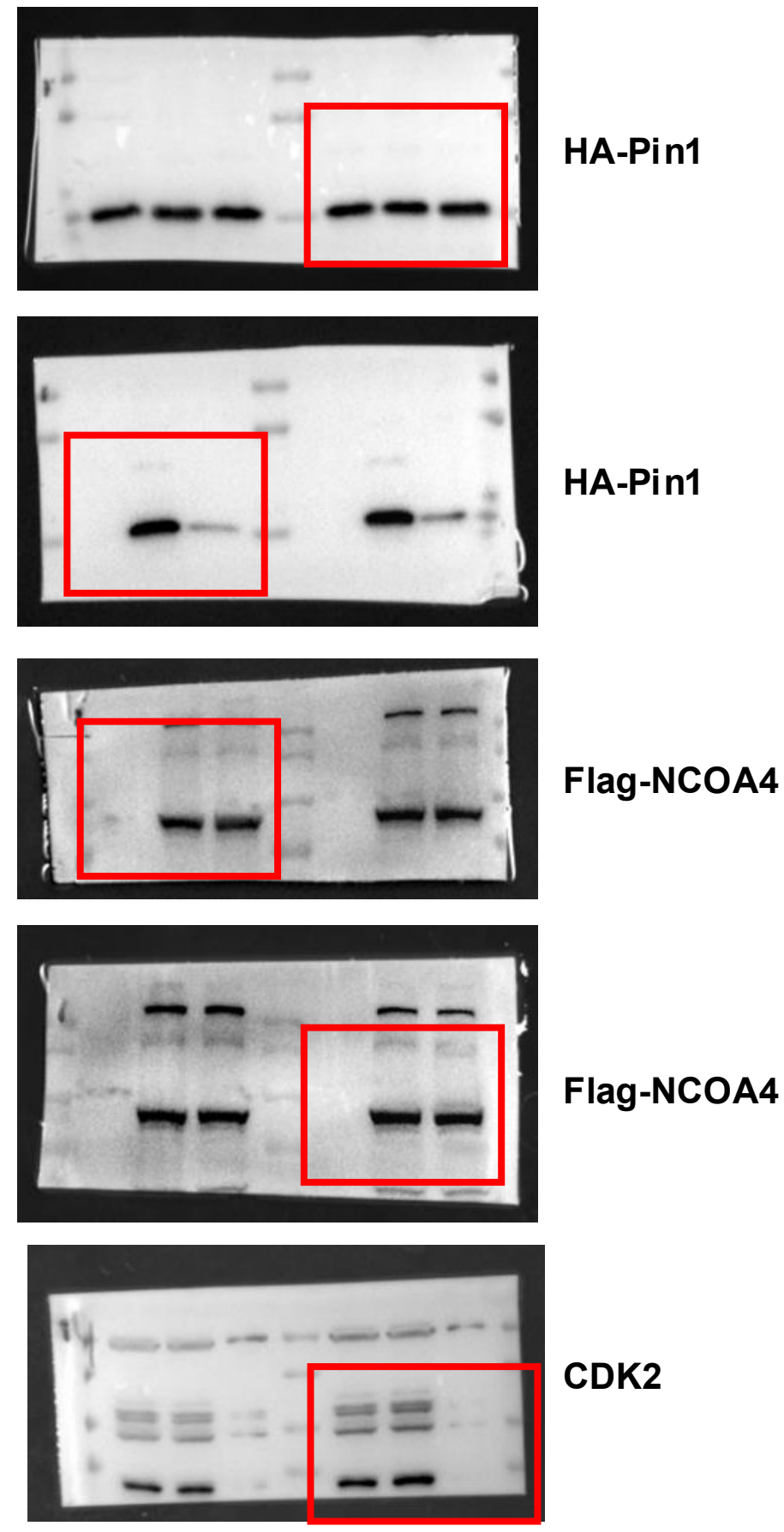

S6G

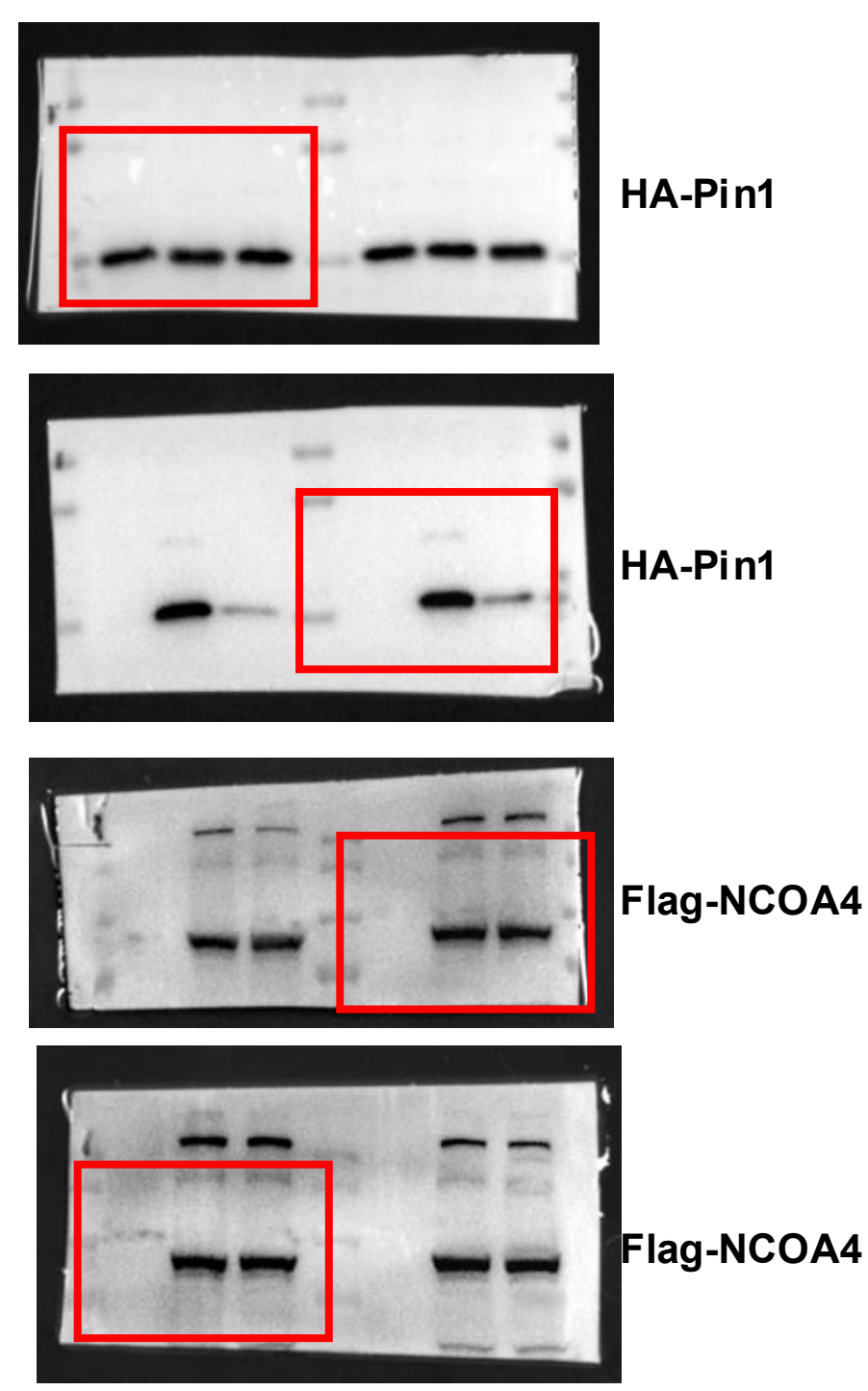

S6H

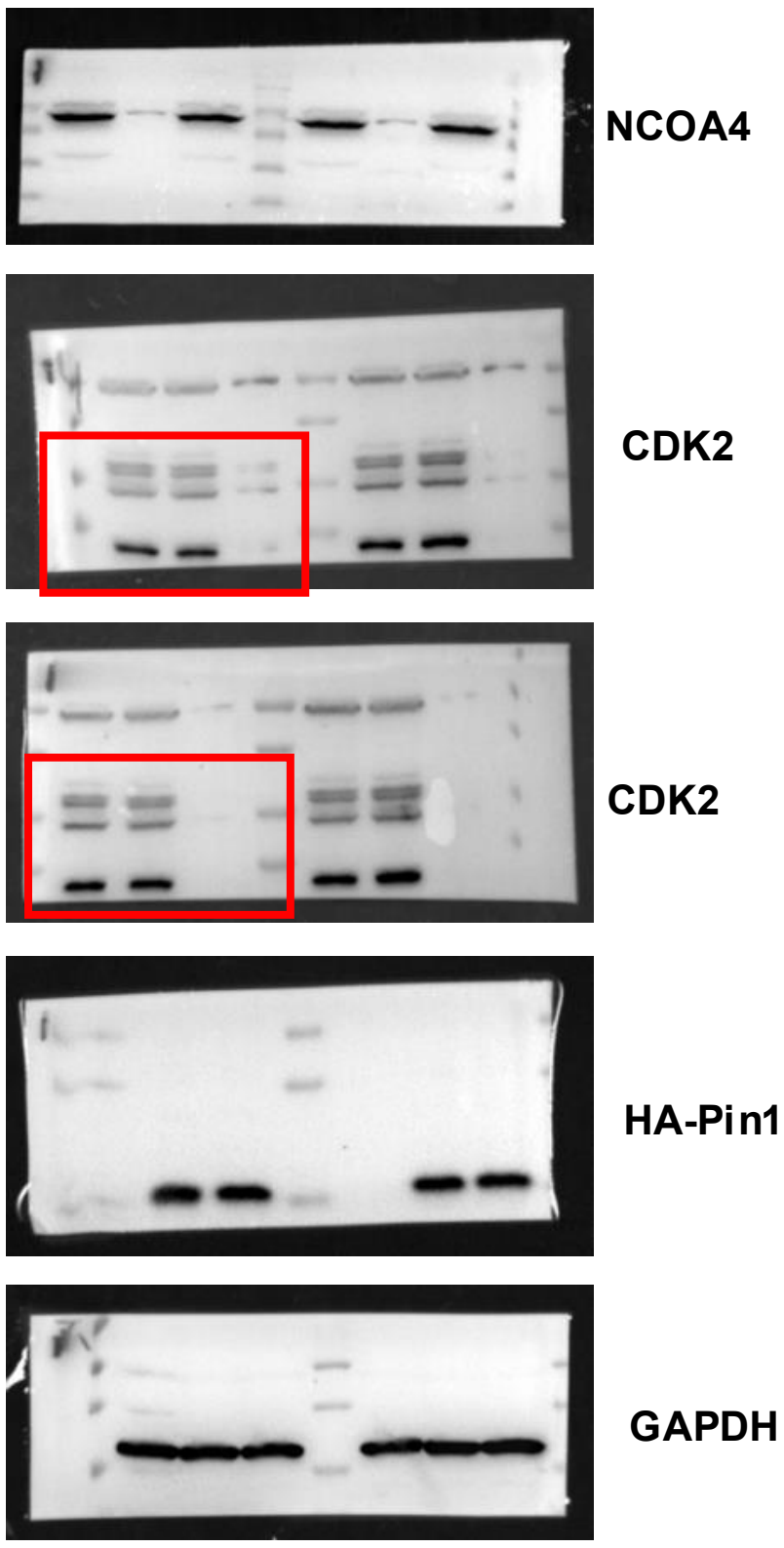

S6I

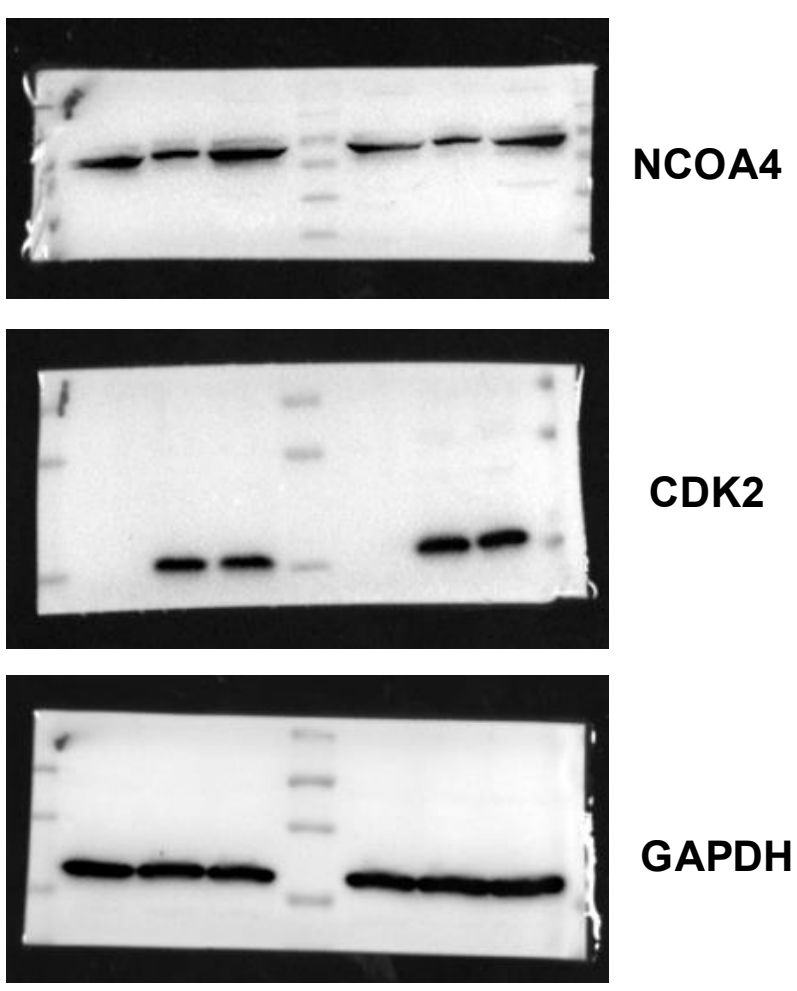

S6J

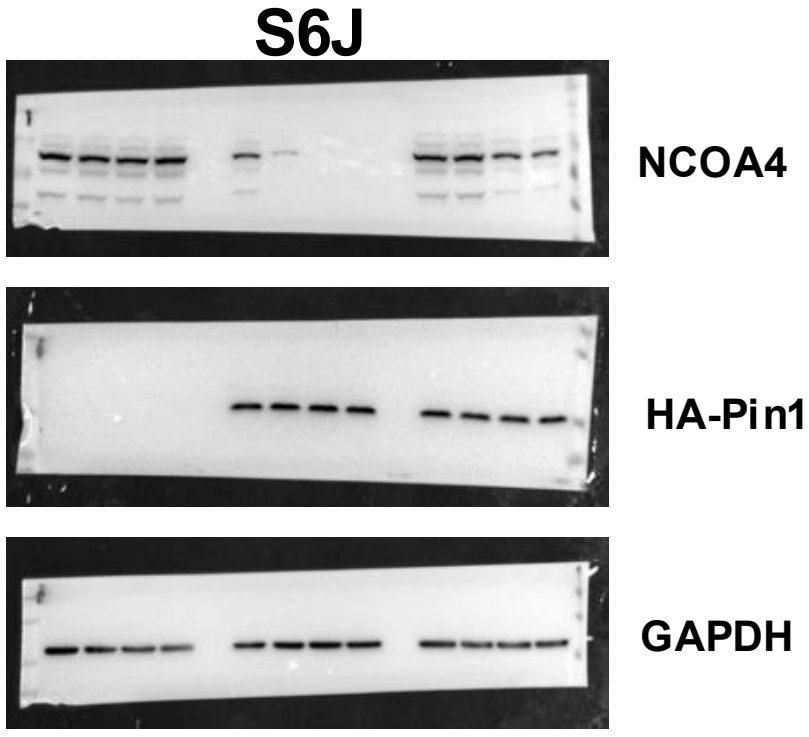

S6L

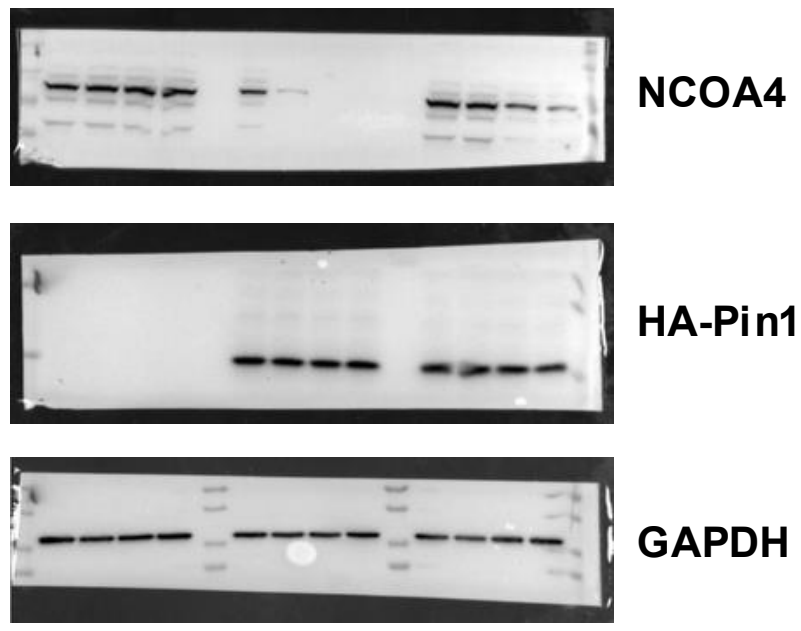

S6M

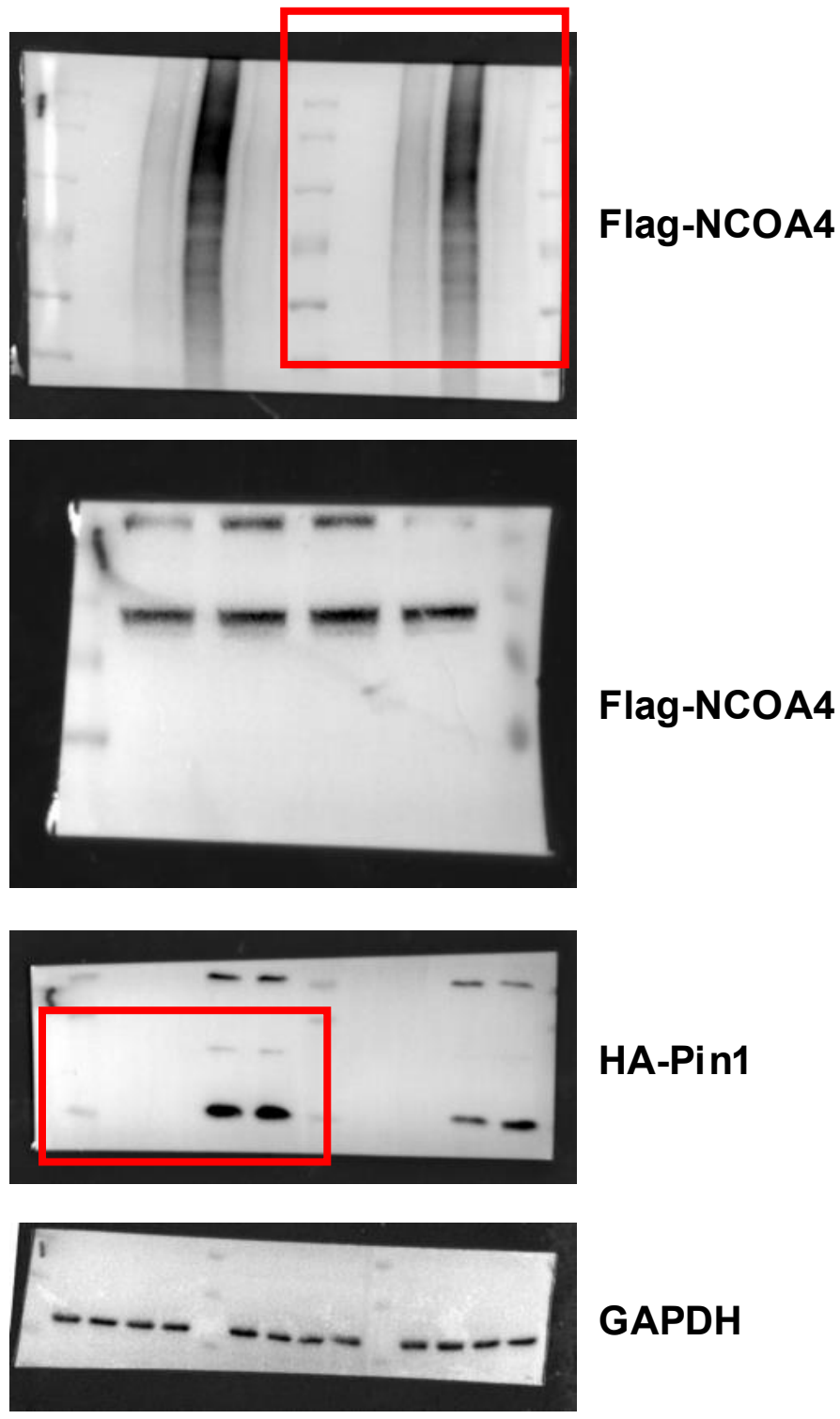

S8A

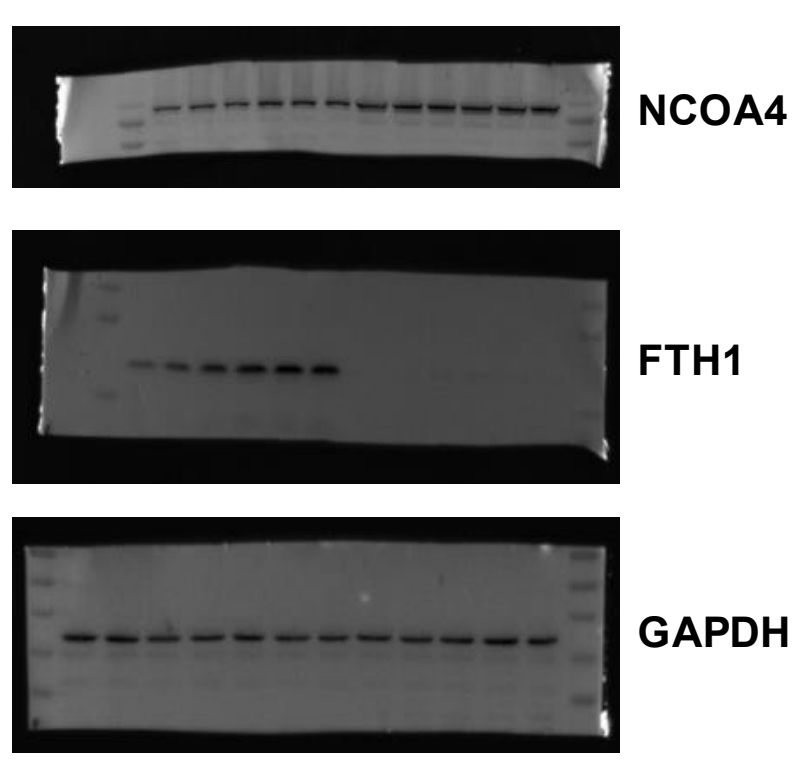

Supplement: Supplementary file 1 [file 41419_2026_9058_MOESM2_ESM.pdf]
