# Supplementary material for: Pin1 targets phosphorylated NCOA4 for K29/K48-linked ubiquitination to suppress ferritinophagy and ferroptosis in anaplastic thyroid cancer
Source: Cell Death Dis. 2026 Jul 1;17(1):815. doi: 10.1038/s41419-026-09058-5 (PMC13586214; doi:10.1038/s41419-026-09058-5)
Supplement: Supplementary file 2 [file 41419_2026_9058_MOESM1_ESM.docx]

**Fig. S1. Pin1 promotes ATC cell growth and tumor progression. A.** Immunoblot analysis of Pin1 protein levels in normal thyroid cell line (N-thy), various papillary thyroid cancer cell lines (TPC-1, KTC-1) and various anaplastic thyroid cancer cell lines (CAL62, ACT-1, KHM5M, 8305C). **B.** Immunoblot analysis of Pin1 protein levels in paired normal thyroid tissues (Normal), papillary thyroid cancer tissues (PTC) and anaplastic thyroid cancer tissues (ATC) from patients. **C.** Representative IHC staining of Pin1 in normal thyroid tissue and ATC tissue. Scale bar, 50 μm. **D.** Quantification of Pin1 H-scores in normal thyroid tissues, papillary thyroid cancer (PTC), and anaplastic thyroid cancer (ATC) samples. Data are presented as mean ± s.d. (n = 12 per group). ****P < 0.0001 by one-way ANOVA test. **E.** Kaplan-Meier survival analysis showing correlation between Pin1 expression and overall survival in ATC patients. High Pin1 expression correlates with poor prognosis. Log-rank test. **F.** Representative images of colony formation assays using control (shCtrl) and Pin1-depleted (shPin1) KHM5M and 8305C cells cultured for 12 days. **G-H.** Quantification of colony numbers from F for KHM5M (G) and 8305C (H) cells. Data are mean ± s.d. (n = 3). ****P < 0.0001 by one-way ANOVA test. **I-J.** Cell viability of control and Pin1-depleted KHM5M (I) and 8305C (J) cells assessed by CCK-8 assay over 3 days. Data are mean ± s.d. (n = 3). ***P < 0.001, ****P < 0.0001 by unpaired two-tailed Student's t-test. **K-M.** Xenograft tumor growth in nude mice injected with control or Pin1-depleted KHM5M cells. Representative images of dissected tumors (K), tumor weights (L), and tumor growth curves (M). Data are mean ± s.d. (n = 7 mice per group). *P < 0.05, ****P < 0.0001 by unpaired t-test (L) or unpaired two-tailed Student's t-test (M).

**Fig. S2. Pin1 depletion promotes ferritinophagy and ferroptosis in ATC cells. A-B.** LDH release from control and Pin1-depleted KHM5M (A) and 8305C (B) cells. Data are mean ± s.d. (n = 3). **P < 0.01, ***P < 0.001 by one-way ANOVA test. **C-D.** Intracellular MDA levels in control and Pin1-depleted KHM5M (C) and 8305C (D) cells. Data are mean ± s.d. (n = 3). ***P < 0.001, ****P < 0.0001 by one-way ANOVA test. **E-F.** Intracellular H₂O₂ levels in control and Pin1-depleted KHM5M (E) and 8305C (F) cells. Data are mean ± s.d. (n = 3). ***P < 0.001, ****P < 0.0001 by one-way ANOVA test. **G-H.** Intracellular Fe²⁺ levels in control and Pin1-depleted KHM5M (G) and 8305C (H) cells. Data are mean ± s.d. (n = 3). ***P < 0.001, ****P < 0.0001 by one-way ANOVA test. **I.** Transmission electron microscopy images showing mitochondrial morphology in control (shCtrl) and Pin1-depleted (shPin1) KHM5M cells. White boxes highlight mitochondria. Scale bars, 1 μm (upper panels), 0.5 μm (lower panels). **J.** Immunoblot analysis of ferroptosis and ferritinophagy-related markers (NCOA4, FTH1, GPX4, ATG7, SQSTM1, LC3) in control and Pin1-depleted KHM5M and 8305C cells. **K.** Representative IHC staining of FTH1 and Ki67 in xenograft tumor tissues from control or Pin1-depleted KHM5M cells. Scale bar, 50 μm.

**Fig. S3. Pin1 promotes NCOA4 degradation in a catalytic activity-dependent manner. A.** Endogenous co-immunoprecipitation assay showing the interaction between Pin1 and NCOA4 in KHM5M cells treated with or without 1 μM erastin. Cell lysates were immunoprecipitated with anti-NCOA4 antibody and analyzed by immunoblotting with the indicated antibodies. **B.** Endogenous co-immunoprecipitation assay showing the interaction between Pin1 and NCOA4 in KHM5M cells treated with or without 1 μM RSL3. Cell lysates were immunoprecipitated with anti-NCOA4 antibody and analyzed by immunoblotting with the indicated antibodies. **C.** Immunoblot analysis of NCOA4 protein levels in KHM5M and 8305C cells overexpressing Pin1-WT or catalytically inactive mutant (W34A/K63A). **D.** Immunoblot analysis of NCOA4 protein levels in KHM5M and 8305C cells treated with DMSO or Pin1 inhibitor KPT-6566 (1 μM) for 24 h. **E.** Cycloheximide chase assay in KHM5M cells overexpressing Pin1-WT or catalytically inactive mutant (W34A/K63A). Representative immunoblots showing NCOA4 protein levels at indicated time points after CHX treatment (100 μg/ml). **F.** Quantification of NCOA4 protein half-life in E. Data are mean ± s.d. (n = 3). ****P < 0.0001 by two-way ANOVA test. **G.** Cycloheximide chase assay in 8305C cells overexpressing Pin1-WT or catalytically inactive mutant (W34A/K63A). Representative immunoblots showing NCOA4 protein levels at indicated time points after CHX treatment (100 μg/ml). **H.** Quantification of NCOA4 protein half-life in G. Data are mean ± s.d. (n = 3). ****P < 0.0001 by two-way ANOVA test. **I.** Cycloheximide chase assay in KHM5M cells treated with KPT-6566 (1 μM) for 24 h. Representative immunoblots showing NCOA4 protein levels at indicated time points after CHX treatment (100 μg/ml). **J.** Quantification of NCOA4 protein half-life in I. Data are mean ± s.d. (n = 3). ****P < 0.0001 by two-way ANOVA test. **K.** Cycloheximide chase assay in 8305C cells treated with KPT-6566 (1 μM) for 24 h. Representative immunoblots showing NCOA4 protein levels at indicated time points after CHX treatment (100 μg/ml). **L.** Quantification of NCOA4 protein half-life in K. Data are mean ± s.d. (n = 3). ****P < 0.0001 by two-way ANOVA test.

**Fig. S4. NCOA4 is required for Pin1 depletion–induced ferritinophagy and ferroptosis in ATC cells. A-B.** LDH release from control or Pin1-depleted KHM5M (A) and 8305C (B) cells transfected with siCtrl or siNCOA4. Data are mean ± s.d. (n = 3). ^ns^P > 0.05, **P < 0.01, ***P < 0.001, ****P < 0.0001 by one-way ANOVA test. **C-D.** Intracellular MDA levels in control or Pin1-depleted KHM5M (C) and 8305C (D) cells transfected with siCtrl or siNCOA4. Data are mean ± s.d. (n = 3). ****P < 0.0001 by one-way ANOVA test. **E-F.** Intracellular H₂O₂ levels in control or Pin1-depleted KHM5M (E) and 8305C (F) cells transfected with siCtrl or siNCOA4. Data are mean ± s.d. (n = 3). ****P < 0.0001 by one-way ANOVA test. **G-H.** Intracellular Fe²⁺ levels in control or Pin1-depleted KHM5M (G) and 8305C (H) cells transfected with siCtrl or siNCOA4. Data are mean ± s.d. (n = 3). ***P < 0.001, ****P < 0.0001 by one-way ANOVA test. **I-J.** Flow cytometric analysis of lipid peroxidation in control or Pin1-depleted KHM5M (I) and 8305C (J) cells transfected with siCtrl or siNCOA4 using C11-BODIPY 581/591. **K.** Mitochondrial membrane potential (ΔΨm) assessed by JC-1 staining in control or Pin1-depleted KHM5M cells transfected with siCtrl or siNCOA4. Representative confocal images showing JC-1 aggregates (red) and monomers (green). Scale bar, 50 μm. **L.** Quantification of the relative JC-1 red/green fluorescence ratio in KHM5M cells shown in K. Data are mean ± s.d. (n = 3). ****P < 0.0001 by one-way ANOVA test. **M.** Immunoblot analysis of ferritinophagy-related markers (NCOA4, FTH1, ATG7, SQSTM1, LC3, GPX4) in control or Pin1-depleted KHM5M and 8305C cells transfected with siCtrl or siNCOA4.

**Fig. S5. Genetic or pharmacologic inhibition of ferritinophagy attenuates the enhanced ferroptosis sensitivity caused by Pin1 depletion in ATC cells. A.** Cell viability of control or Pin1-depleted KHM5M (top) and 8305C (bottom) cells transfected with siCtrl or siNCOA4 and treated with the indicated concentrations of erastin for 24 h, assessed by CCK-8 assay. Data are mean ± s.d. (n = 3). ***P < 0.001, ****P < 0.0001 by two-way ANOVA test. **B.** Representative images of colony formation assays using control or Pin1-depleted KHM5M (left) and 8305C (right) cells transfected with siCtrl or siNCOA4 and treated with DMSO or erastin (1 μM or 3 μM) for 12 days. **C.** Quantification of colony numbers from B for KHM5M (top) and 8305C (bottom) cells. Data are mean ± s.d. (n = 3). ^ns^P ＞ 0.05, **P < 0.01, ***P < 0.001, ****P < 0.0001 by two-way ANOVA with Sidak’s post hoc test. **D.** Cell viability of control or Pin1-depleted KHM5M (top) and 8305C (bottom) cells treated with or without 20 μM chloroquine (CQ) and exposed to the indicated concentrations of erastin for 24 h, assessed by CCK-8 assay. Data are mean ± s.d. (n = 3). ***P < 0.001, ****P < 0.0001 by two-way ANOVA test. **E.** Representative images of colony formation assays using control or Pin1-depleted KHM5M (left) and 8305C (right) cells treated with DMSO or erastin (1 μM or 3 μM) in the presence or absence of 20 μM CQ for 12 days. **F.** Quantification of colony numbers from E for KHM5M (top) and 8305C (bottom) cells. Data are mean ± s.d. (n = 3). ^ns^P ＞ 0.05, *P < 0.05, ***P < 0.001 by two-way ANOVA with Sidak’s post hoc test.

**Fig. S6. CDK2 phosphorylates NCOA4 at Ser572 to regulate its stability. A.** Co-immunoprecipitation assay showing endogenous interaction between CDK2 and NCOA4 in ATC patient tissues. IgG served as negative control. **B.** Schematic representation of the conserved CDK2 phosphorylation consensus motif (pS-P) at NCOA4-Ser572 across species. **C.** Dot blot immunoblot assay validating anti-NCOA4-pS572 phospho-specific antibody using synthetic peptides. Non-phosphorylated (WT) and phosphorylated (Phospho) peptides were spotted at indicated doses (0.01, 0.03, 0.10, 0.30 μg). Membranes were probed with anti-pNCOA4-S572 or anti-NCOA4 antibodies. **D.** Immunoblot analysis of NCOA4-pS572 in KHM5M and 8305C cells treated with DMSO (vehicle control) or CDK inhibitors for 12 h: Ro-3306 (CDK1 inhibitor, 1 μM), CVT-313 (CDK2 inhibitor, 1 μM), ribociclib (CDK4/6 inhibitor, 1 μM), or abemaciclib (CDK4/6 inhibitor, 1 μM). **E.** Immunoblot analysis of NCOA4-pS572 in control or CDK2-depleted KHM5M and 8305C cells. **F.** Flag-immunoprecipitation assay showing that CDK2 depletion reduces interaction between Pin1 and NCOA4 in HEK-293T cells co-transfected with Flag-NCOA4 and HA-Pin1. **G.** Flag-immunoprecipitation assay showing that CDK2 inhibitor CVT-313 (1 μM, 12 h) reduces interaction between Pin1 and NCOA4 in HEK-293T cells. **H.** Immunoblot analysis of NCOA4 protein levels in KHM5M and 8305C cells overexpressing Pin1 with or without CDK2 knockdown. **I.** Immunoblot analysis of NCOA4 protein levels in KHM5M and 8305C cells overexpressing Pin1 treated with DMSO or CVT-313 (1 μM) for 12 h. **J.** Cycloheximide chase assay in Pin1-overexpressing KHM5M cells treated with DMSO or CVT-313 (1 μM). Cells were treated with CHX (100 μg/ml) for indicated time points. **K.** Quantification of NCOA4 protein half-life in J. Data are mean ± s.d. (n = 3). ***P < 0.001, ****P < 0.0001 by two-way ANOVA test. **L.** Cycloheximide chase assay in Pin1-overexpressing 8305C cells treated with DMSO or CVT-313 (1 μM). Cells were treated with CHX (100 μg/ml) for indicated time points. **M.** Quantification of NCOA4 protein half-life in L. Data are mean ± s.d. (n = 3). ****P < 0.0001 by two-way ANOVA test. **N.** In vivo ubiquitination assay showing that CDK2 inhibitor CVT-313 reduces Pin1-mediated NCOA4 ubiquitination. HEK-293T cells were co-transfected with Flag-NCOA4, His-Ub, and HA-Pin1, treated with CVT-313 (1 μM) and MG132 (10 μM) for 12 h before harvest.

**Fig. S7. NCOA4 phosphorylation at Ser572 inhibits ferroptosis and promotes ATC tumor growth. A.** Cell viability of KHM5M (top) and 8305C (bottom) cells stably expressing NCOA4-WT, S572A, or S572D treated with indicated concentrations of erastin for 24 h, assessed by CCK-8 assay. Data are mean ± s.d. (n = 3). ****P < 0.0001 by two-way ANOVA test. **B.** Representative images of colony formation assays using KHM5M and 8305C cells expressing NCOA4 variants treated with DMSO or erastin (1 μM or 3 μM) for 12 days. **C.** Quantification of colony numbers from B for KHM5M (top) and 8305C (bottom) cells. Data are mean ± s.d. (n = 3). ^ns^P > 0.05, **P < 0.01, ****P < 0.0001 by two-way ANOVA test. **D-F.** Xenograft tumor growth in nude mice injected with KHM5M cells expressing NCOA4 variants. Representative images of dissected tumors (D), tumor weights (E), and tumor growth curves (F). Data are mean ± s.d. (n = 8 mice per group). ****P < 0.0001 by one-way ANOVA test (E) or unpaired two-tailed Student's t-test (F). **G.** Representative IHC staining of FTH1, Ki67, pNCOA4 and NCOA4 in xenograft tumor tissues from D. Scale bar, 50 μm.

**Fig. S8. Pin1 inhibitor KPT-6566 promotes ferroptosis in vivo with minimal systemic toxicity. A.** Immunoblot analysis of NCOA4 and FTH1 in tumor lysates from xenografts treated with DMSO or KPT-6566. GAPDH served as the loading control. **B.** Representative immunohistochemical staining of NCOA4, FTH1, and Ki67 in xenograft tumors from mice treated with DMSO or KPT-6566. Scale bar, 200 μm. **C.** Representative H&E staining images of major organs, including heart, liver, spleen, lung, and kidney, from mice treated with DMSO or KPT-6566, showing no obvious histopathological abnormalities under the indicated treatment conditions. Scale bar, 200 μm.
